# Stress-Strength Reliability for Exponentiated Inverted Weibull Distribution with Application on Breaking of Jute Fiber and Carbon Fibers

**DOI:** 10.1155/2021/4227346

**Published:** 2021-09-21

**Authors:** Wael S. Abu El Azm, Ehab M. Almetwally, Abdulaziz S. Alghamdi, Hassan M. Aljohani, Abdisalam Hassan Muse, O. E. Abo-Kasem

**Affiliations:** ^1^Department of Statistics, Faculty of Commerce, Zagazig University, Zagazig, Egypt; ^2^Department of Statistics, Faculty of Business Administration, Delta University of Science and Technology, Gamasa, Egypt; ^3^Department of Mathematics, College of Science & Arts, King Abdulaziz University, P.O. Box 344, Rabigh 21911, Saudi Arabia; ^4^Department of Mathematics & Statistics, College of Science, Taif University, P.O. Box 11099, Taif 21944, Saudi Arabia; ^5^Pan African University, Institute of Basic Science, Technology and Innovation (PAUSTI), Nairobi 6200-00200, Kenya

## Abstract

For the first time and by using an entire sample, we discussed the estimation of the unknown parameters *θ*_1_, *θ*_2_, and *β* and the system of stress-strength reliability *R*=*P*(*Y* < *X*) for exponentiated inverted Weibull (EIW) distributions with an equivalent scale parameter supported eight methods. We will use maximum likelihood method, maximum product of spacing estimation (MPSE), minimum spacing absolute-log distance estimation (MSALDE), least square estimation (LSE), weighted least square estimation (WLSE), method of Cramér-von Mises estimation (CME), and Anderson-Darling estimation (ADE) when *X* and *Y* are two independent a scaled exponentiated inverted Weibull (EIW) distribution. Percentile bootstrap and bias-corrected percentile bootstrap confidence intervals are introduced. To pick the better method of estimation, we used the Monte Carlo simulation study for comparing the efficiency of the various estimators suggested using mean square error and interval length criterion. From cases of samples, we discovered that the results of the maximum product of spacing method are more competitive than those of the other methods. A two real‐life data sets are represented demonstrating how the applicability of the methodologies proposed in real phenomena.

## 1. Introduction

Since Birnbaum's [1] pioneering research, statistical inference of a system stress-strength parameter has received increased attention and is widely used in a variety of engineering applications. If *X* and *Y* are two independent random variables that represent the strength and the stress, then *R*=*P*(*Y* < *X*) is a measure of system performance that naturally arises in mechanical dependability. In this case, the system fails if and only if the applied stress is greater than its strength at any time. Several studies in the statistical literature have investigated the problem of estimating the stress-strength parameters of a system. Kundu and Gupta [2, 3] introduced the estimation of stress-strength reliability for generalized exponential and Weibull random variables, respectively. um likelihood method, max Raqab et al. [4] discussed the estimation of *R*, where *X* and *Y* are distributed as two independent three-parameter generalized exponential random variables. Rezaei et al. [5] considered the estimation of *R*, where *X* and *Y* are two independent generalized Pareto distributions.

Recently, many authors investigated the estimation of *R*=*P*(*Y* < *X*) for different life testing schemes based on different distributions by using the maximum likelihood and Bayesian estimation methods; see, for example, Mokhlis [6], Kundu and Raqab [7], Asgharzadeh et al. [8], Asgharzadeh et al. [9], Asgharzadeh et al. [10], Valiollahi et al. [11], Rao et al. [12], Rao et al. [13], Mirjalili et al. [14], Jia et al. [15], Nadeb et al. [16], Alshenawy et al. [17], El-Sherpieny et al. [18], Nassr et al. [19], and Muhammad et al. [20]. As frequentist methods, the maximum likelihood method and the Bayesian estimation method were used in these studies. However, little thought was given to estimate *R*=*P*(*Y* < *X*) using other methods, although, in some cases, they can provide better estimates than the maximum likelihood approach. Almetwally and Almongy [21] examined classical and Bayesian estimation methods for the stress-strength model of the power Lomax distribution.

Aside from the maximal likelihood estimation (MLE) approach, Almarashi et al. [22] offered nine other frequentist estimate methods to estimate the stress-strength reliability of the Weibull distribution, namely, least square, weighted least square, percentile, maximum product of spacing, minimum spacing absolute distance, minimum spacing absolute-log distance, method of Cramér-von Mises, and Anderson-Darling and right-tail Anderson-Darling. They compared the efficiency of the different proposed estimators by using Monte Carlo simulation study. In terms of relative biases and relative mean squared errors, the performance and finite sample properties of the various estimators are compared.

To the authors' knowledge, the MLE and the maximum product of spacing method which were used to estimate the parameters *θ*_1_, *θ*_2_, and *β* of life of an EIW under a finite sample (MPSE), minimum spacing absolute-log distance estimation (MSALDE) method, least square estimation (LSE) method, weighted least square estimation (WLSE) method of Cramér-von Mises estimation (CME), and Anderson-Darling estimation (ADE) method have not yet been investigated. In this paper, our main purpose is to use eight approaches to derive estimates of the unknown parameters and stress-strength reliability *R*=*P*(*Y* < *X*), which we think if applied by statisticians/reliability engineers would be very interesting in a scaled exponentiated inverted Weibull distribution. Furthermore, the simulation study and real data analysis demonstrate that there are classical methods, rather than MLE methods, which can provide desirable estimates, justifying their use in applied areas. It should also be noted that this is the first time that eight estimation methods have been considered to estimate the unknown parameters *θ*_1_, *θ*_2_, and *β* and stress-strength reliability *R*=*P*(*Y* < *X*) of the EIW distribution.

Flaih et al. [23] proposed the exponentiated inverted Weibull distribution as a generalization of the standard parent distribution known as the exponentiated-parent distribution and the standard inverted Weibull distribution. More research on the EIW distribution is needed, both theoretically (estimation methods) and practically (analysis further data). According to this study, the EIW can be used as an alternative to the inverted Weibull distribution and may perform better than the inverted Weibull distribution. For *θ*=1, it represents the standard inverted Weibull distribution, and for *β*=1, it represents the exponentiated standard inverted exponential distribution. As a result, the exponentiated inverted Weibull distribution is a generalization of both the exponentiated inverted exponential and inverted Weibull distributions. The physical interpretation of the exponentiated inverted Weibull distribution is also available.

The EIW with scale parameter *θ* and shape parameter *β*, denoted by EIW (*θ*, *β*), has the following probability density function (PDF):

(1) $$\begin{array}{c} f\left(x;\theta ,\beta \right)=\theta \beta x^{-\left(\beta +1\right)}\exp\;{\left[-x^{-\beta }\right]}^{\theta },\;x>0,\alpha ,\beta >0, \end{array}$$

and the corresponding cumulative distribution function (CDF) is given by

(2) $$\begin{array}{c} F\left(x;\theta ,\beta \right)=\exp\;{\left[-x^{-\beta }\right]}^{\theta },\;x>0,\theta ,\beta >0. \end{array}$$

Let *X* and *Y* be independent exponentiated inverted Weibull random variables and follow EIW (*θ*_1_, *β*) and EIW (*θ*_2_, *β*), respectively; then *R*=*P*(*Y* < *X*) can be written as follows (see Hassan et al. [24]):

(3) $$\begin{array}{c} R=\frac{\theta_{1}}{\theta_{1}+\theta_{2}}. \end{array}$$

**Figure 1:**
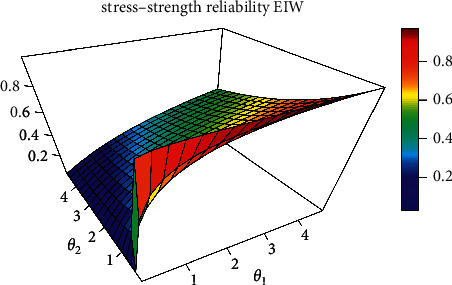
3 dimensions of stress-strength reliability for EIW distribution with different parameters.

Figure 1 shows different values for *R* when *θ*_1_ and *θ*_2_ change.

The main objective of this study is to estimate unknown parameters *θ*_1_, *θ*_2_, and *β* and stress-strength reliability *R*=*P*(*Y* < *X*) when *X* and *Y* are independent of a scaled EIW distribution using the eight estimation methods listed above. Furthermore, for the stress-strength parameter, we use percentile bootstrap and bias-corrected percentile bootstrap confidence intervals. To compare the efficiency of the various estimates, we conduct an extensive Monte Carlo numerical simulation study, as well as an analysis of two real‐life data sets, the applicability of the methodologies proposed in real phenomena. The rest of this paper is organized as follows: In Section 2, we proposed the different estimation methods. Percentile bootstrap and bias-corrected percentile bootstrap confidence intervals are discussed in Section 3. In Section 4, a Monte Carlo numerical simulation research is carried out. In Section 5, two real-life data sets are examined. Finally, Section 6 concludes the paper.

## 2. Different Estimation Methods

In this section, the eight recurrent estimation methods considered in this paper to obtain the unknown parameters and different estimates of the stress-strength parameter will be discussed. These estimation methods would be of particular interest when comparing them with other maximum likelihood estimation procedures. For more examples of classical estimation method, see the works of Almetwally [25], El-Morshedy et al. [26], Almetwally et al. [27], and Sabry et al. [28].

### 2.1. Maximum Likelihood Estimation

Let *x*_1_, *x*_2_,…, *x*_*n*_ and *y*_1_, *y*_2_,…, *y*_*k*_ be random samples from EIW (*θ*_1_, *β*) and EIW (*θ*_2_, *β*), respectively, and the likelihood function of the observed sample can be expressed as

(4) $$\begin{array}{c} L\left(\theta_{1},\theta_{2},\beta \right)=\prod_{i=1}^{n}\theta_{1}\beta x_{i}^{-\left(\beta +1\right)}\exp\;{\left[-x_{i}^{-\beta }\right]}^{\theta_{1}}\prod_{j=1}^{k}\theta_{2}\beta y_{j}^{-\left(\beta +1\right)}\exp\;{\left[-y_{j}^{-\beta }\right]}^{\theta_{2}}. \end{array}$$

We obtain *l*=log  *L*(*θ*_1_, *θ*_2_, *β*) by taking the natural logarithm likelihood function as

(5) $$\begin{array}{c} l=\left(n+k\right)\log\;\beta +n\;\log\;\theta_{1}+k\;\log\;\theta_{2}-\left(\beta +1\right)\left[\sum_{i=1}^{n}\log\;x_{i}+\sum_{j=1}^{k}\log\;y_{j}\right]-\theta_{1}\sum_{i=1}^{n}x_{i}^{-\beta }-\theta_{2}\sum_{j=1}^{k}y_{j}^{-\beta }. \end{array}$$

The MLEs of *θ*_1_, *θ*_2_, and *β* denoted by ${\hat{\theta }}_{1}^{\text{MLE}}$, ${\hat{\theta }}_{2}^{\text{MLE}}$, and ${\hat{\beta }}^{MLE}$, respectively, can be obtained by solving the subsequent equations:

(6) $$\begin{array}{c} \frac{\partial l}{\partial \theta_{1}}=\frac{n}{\theta_{1}}-\sum_{i=1}^{n}x_{i}^{-\beta }, \end{array}$$

(7) $$\begin{array}{c} \frac{\partial l}{\partial \theta_{2}}=\frac{k}{\theta_{2}}-\sum_{j=1}^{k}y_{i}^{-\beta }, \end{array}$$

(8) $$\begin{array}{c} \frac{\partial l}{\partial \beta }=\frac{\left(n+k\right)}{\beta }\;-\sum_{i=1}^{n}\log\;x_{i}-\sum_{j=1}^{k}\log\;y_{j}+\theta_{1}\sum_{i=1}^{n}x_{i}^{-\beta }\log\left(x_{i}\right)+\theta_{2}\sum_{j=1}^{k}y_{j}^{-\beta }\log\left(y_{j}\right). \end{array}$$

${\hat{\theta }}_{1}^{\text{MLE}}$ and ${\hat{\theta }}_{2}^{\text{MLE}}$ can be obtained as a function of the unknown parameter *β* from (6) and (7), respectively, as follows:

(9) $$\begin{array}{c} {\hat{\theta }}_{1}^{\text{MLE}}\left(\beta \right)=\frac{n}{\sum_{i=1}^{n}x_{i}^{-\beta }},\\ {\hat{\theta }}_{2}^{\text{MLE}}\left(\beta \right)=\frac{k}{\sum_{j=1}^{n}y_{i}^{-\beta }}. \end{array}$$

Substituting the estimators *θ*_1_^MLE^(*β*) and *θ*_2_^MLE^(*β*) obtained from (9) in (5), the profile log-likelihood function of parameter *β* can then be obtained as follows:

(10) $$\begin{array}{c} l=\left(n+k\right)\log\;\beta -\beta \left[\sum_{i=1}^{n}\log\;x_{i}+\sum_{j=1}^{k}\log\;y_{j}\right]-n\;\log\left(\sum_{i=1}^{n}x_{i}^{-\beta }\right)-k\;\log\left(\sum_{j=1}^{k}y_{j}^{-\beta }\right). \end{array}$$

To obtain *β*^MLE^, as a result of differentiating (10) with respect to *β* and equating the result by zero,

(11) $$\begin{array}{c} \psi \left(\beta \right)={\left(\frac{\sum_{i=1}^{n}\log\;x_{i}+\sum_{j=1}^{n}\log\;y_{i}}{\left(n+k\right)}-\frac{n\sum_{i=1}^{n}x_{i}^{-\beta }\log\left(x_{i}\right)}{\left(n+k\right)\sum_{i=1}^{n}x_{i}^{-\beta }}-\frac{k\sum_{j=1}^{n}y_{i}^{-\beta }\log\left(y_{j}\right)}{\left(n+k\right)\sum_{j=1}^{n}y_{i}^{-\beta }}\right)}^{-1}. \end{array}$$

After obtaining ${\hat{\beta }}^{\text{MLE}}$ from (11) by using any iteration procedure, we can obtain ${\hat{\theta }}_{1}^{\text{MLE}}$ and ${\hat{\theta }}_{2}^{\text{MLE}}$ from (9). Now, the MLE of a system *R* can be obtained as

(12) $$\begin{array}{c} {\hat{R}}^{MLE}=\frac{{\hat{\theta }}_{1}^{MLE}}{{\hat{\theta }}_{1}^{MLE}+{\hat{\theta }}_{2}^{MLE}}\cdot g\left(\lambda_{j}\right)\propto \lambda_{j}^{a_{j}-1}e^{-\lambda_{j}b_{j}},\;a_{j},b_{j}>0,j=1,\;2. \end{array}$$

### 2.2. Maximum Product of Estimation

Cheng and Amin [29] introduced the method of maximum product of spacing as an alternative to the maximum likelihood method to estimate the parameters of the lognormal distribution. Let *x*_1:*n*_, *x*_2:*n*_,…, *x*_*n*:*n*_ denote the order statistics of a random sample *n* from EIW (*θ*_1_, *β*) and let *y*_1:*k*_, *y*_2:*k*_,…, *y*_*k*:*k*_ denote the order statistics of a random sample *k* from EIW (*θ*_2_, *β*); the uniform spacings of the two samples can therefore be defined as follows:

Let *x*_1:*n*_, *x*_2:*n*_,…, *x*_*n*:*n*_ denote the order statistics of a random sample from EIW.

(13) $$\begin{array}{c} \Delta_{1i}=F\left(\frac{x_{i:n}}{\theta_{1}},\beta \right)-F\left(\frac{x_{i-1:n}}{\theta_{1}},\beta \right),\\ \Delta_{2j}=F\left(\frac{y_{j:k}}{\theta_{2}},\beta \right)-F\left(\frac{y_{j-1:k}}{\theta_{2}},\beta \right). \end{array}$$

The MPSEs of the unknown parameters are produced by maximization of the following function, as in the work of Cheng and Amin [30].

(14) $$\begin{array}{c} \text{MP}\left(\theta_{1},\theta_{2},\beta \right)=\frac{1}{n+1}\sum_{i=1}^{n+1}\log\left(\Delta_{1i}\right)+\frac{1}{k+1}\sum_{j=1}^{k+1}\log\left(\Delta_{2j}\right). \end{array}$$

From (2) and (14), the MPSEs of the unknown parameters *θ*_1_, *θ*_2_, and *β* denoted by ${\hat{\theta }}_{1}^{\text{MPSE}}$, ${\hat{\theta }}_{2}^{\text{MPSE}}$, and ${\hat{\beta }}^{\text{MPSE}}$ can be obtained by maximizing, with respect to *θ*_1_, *θ*_2_, and *β*, the following function:

(15) $$\begin{array}{c} \text{MP}\left(\theta_{1},\theta_{2},\beta \right)=\frac{1}{n+1}\sum_{i=1}^{n+1}\log\left[{\left(e^{-x_{i:n}^{-\beta }}\right)}^{\theta_{1}}-{\left(e^{-x_{i-1:n}^{-\beta }}\right)}^{\theta_{1}}\right]+\frac{1}{k+1}\sum_{j=1}^{k+1}\log\left[{\left(e^{-y_{j:k}^{-\beta }}\right)}^{\theta_{2}}-{\left(e^{-y_{j-1:k}^{-\beta }}\right)}^{\theta_{2}}\right]. \end{array}$$

These estimates can be obtained equivalently by solving the following equations simultaneously:

(16) $$\begin{array}{c} \frac{\partial \text{MP}\left(\theta_{1},\theta_{2},\beta \right)}{\partial \theta_{1}}=\frac{1}{n+1}\sum_{i=1}^{n+1}\frac{x_{i:n}^{-\beta }{\left(e^{-x_{i:n}^{-\beta }}\right)}^{\theta_{1}}-x_{i-1:n}^{-\beta }{\left(e^{-x_{i-1:n}^{-\beta }}\right)}^{\theta_{1}}}{{\left(e^{-x_{i:n}^{-\beta }}\right)}^{\theta_{1}}-{\left(e^{-x_{i-1:n}^{-\beta }}\right)}^{\theta_{1}}}=0,\\ \frac{\partial \text{MP}\left(\theta_{1},\theta_{2},\beta \right)}{\partial \theta_{2}}=\frac{1}{k+1}\sum_{j=1}^{k+1}\frac{y_{j:k}^{-\beta }{\left(e^{-y_{j:k}^{-\beta }}\right)}^{\theta_{2}}-y_{j-1:k}^{-\beta }\;{\left(e^{-y_{j-1:k}^{-\beta }}\right)}^{\theta_{2}}}{{\left(e^{-y_{j:k}^{-\beta }}\right)}^{\theta_{2}}-{\left(e^{-y_{j-1:k}^{-\beta }}\right)}^{\theta_{2}}}=0,\\ \frac{\partial \text{MP}\left(\theta_{1},\theta_{2},\beta \right)}{\partial \beta }=\frac{\theta_{1}}{n+1}\sum_{i=1}^{n+1}\frac{{Ζ}_{i:n}-{Ζ}_{i-1:n}}{{\left(e^{-x_{i:n}^{-\beta }}\right)}^{\theta_{1}}-{\left(e^{-x_{i-1:n}^{-\beta }}\right)}^{\theta_{1}}}+\frac{\theta_{2}}{k+1}\sum_{j=1}^{k+1}\frac{\Psi_{j:k}-\Psi_{j-1:k}\;}{{\left(e^{-y_{j:k}^{-\beta }}\right)}^{\theta_{2}}-{\left(e^{-y_{j-1:k}^{-\beta }}\right)}^{\theta_{2}}}=0, \end{array}$$

where Ζ_*i*:*n*_=*x*_*i*:*n*_^−*β*^(*e*^−*x*_*i*:*n*_^−*β*^^)^*θ*_1_^log(*x*_*i*:*n*_) and Ψ_*j*:*k*_=*y*_*j*:*k*_^−*β*^(*e*^−*y*_*j*:*k*_^−*β*^^)^*θ*_2_^log(*y*_*j*:*k*_). Using the obtained estimates, we can obtain the MPSE of a system *R* as

(17) $$\begin{array}{c} {\hat{R}}^{\text{MPSE}}=\frac{{\hat{\theta }}_{1}^{\text{MPSE}}}{{\hat{\theta }}_{1}^{\text{MPSE}}+{\hat{\theta }}_{2}^{\text{MPSE}}}. \end{array}$$

### 2.3. Minimum Spacing Distance Estimation

Torabi [31] was the first to propose the minimum spacing distance estimating method. The minimum spacing distance estimators (MSADEs) are obtained by minimizing the following function, using the same notations as in the previous subsections:

(18) $$\begin{array}{c} \text{MD}\left(\theta_{1},\theta_{2},\beta \right)=\sum_{i=1}^{n+1}\psi \left(\Delta_{1i},\phi_{1}\left(n\right)\right)+\sum_{j=1}^{k+1}\psi \left(\Delta_{2j},\phi_{2}\left(m\right)\right), \end{array}$$

where *ϕ*_1_(*n*)=(1/(*n*+1)), *ϕ*_2_(*m*)=(1/(*m*+1)), and *ψ*(*a*, *b*) is an appropriate distance. The most common selections of *ψ*(*a*, *b*) in (18) are called absolute distance |*a* − *b*| and absolute-log distance |log(*a*) − log(*b*)|. The MSADEs of the unknown parameters denoted by ${\hat{\theta }}_{1}^{\text{MSADE}}$, ${\hat{\theta }}_{2}^{\text{MSADE}}$, and ${\hat{\beta }}^{\text{MSADE}}$ can be determined by minimizing the the next function in terms of *θ*_1_, *θ*_2_, and *β*.

(19) $$\begin{array}{c} \text{MD}\left(\theta_{1},\theta_{2},\beta \right)=\sum_{i=1}^{n+1}\left|{\left(e^{-x_{i:n}^{-\beta }}\right)}^{\theta_{1}}-{\left(e^{-x_{i-1:n}^{-\beta }}\right)}^{\theta_{1}}-\phi_{1}\left(n\right)\right|+\sum_{j=1}^{k+1}\left|{\left(e^{-y_{j:k}^{-\beta }}\right)}^{\theta_{2}}-{\left(e^{-y_{j-1:k}^{-\beta }}\right)}^{\theta_{2}}-\phi_{2}\left(m\right)\right|. \end{array}$$

Simultaneously, the three following equations are solved:

(20) $$\begin{array}{c} \frac{\partial \text{MD}\left(\theta_{1},\theta_{2},\beta \right)}{\partial \theta_{1}}=\sum_{i=1}^{n+1}\frac{{\left(e^{-x_{i:n}^{-\beta }}\right)}^{\theta_{1}}-{\left(e^{-x_{i-1:n}^{-\beta }}\right)}^{\theta_{1}}-\phi_{1}\left(n\right)}{\left|{\left(e^{-x_{i:n}^{-\beta }}\right)}^{\theta_{1}}-{\left(e^{-x_{i-1:n}^{-\beta }}\right)}^{\theta_{1}}-\phi_{1}\left(n\right)\right|}\left(x_{i:n}^{-\beta }{\left(e^{-x_{i:n}^{-\beta }}\right)}^{\theta_{1}}-x_{i-1:n}^{-\beta }{\left(e^{-x_{i-1:n}^{-\beta }}\right)}^{\theta_{1}}\right)=0,\\ \frac{\partial \text{MD}\left(\theta_{1},\theta_{2},\beta \right)}{\partial \theta_{2}}=\sum_{j=1}^{m+1}\frac{{\left(e^{-y_{j:k}^{-\beta }}\right)}^{\theta_{2}}-{\left(e^{-y_{j-1:k}^{-\beta }}\right)}^{\theta_{2}}-\phi_{2}\left(m\right)}{\left|{\left(e^{-y_{j:k}^{-\beta }}\right)}^{\theta_{2}}-{\left(e^{-y_{j-1:k}^{-\beta }}\right)}^{\theta_{2}}-\phi_{2}\left(m\right)\right|}\left(y_{j:k}^{-\beta }{\left(e^{-y_{j:k}^{-\beta }}\right)}^{\theta_{2}}-y_{j-1:k}^{-\beta }{\left(e^{-y_{j-1:k}^{-\beta }}\right)}^{\theta_{2}}\right)=0,\\ \frac{\partial \text{MD}\left(\theta_{1},\theta_{2},\beta \right)}{\partial \beta }=\theta_{1}\sum_{i=1}^{n+1}\frac{{\left(e^{-x_{i:n}^{-\beta }}\right)}^{\theta_{1}}-{\left(e^{-x_{i-1:n}^{-\beta }}\right)}^{\theta_{1}}-\phi_{1}\left(n\right)}{\left|{\left(e^{-x_{i:n}^{-\beta }}\right)}^{\theta_{1}}-{\left(e^{-x_{i-1:n}^{-\beta }}\right)}^{\theta_{1}}-\phi_{1}\left(n\right)\right|}\left(Z_{i:n}-Z_{i-1:n}\right)\\ +\theta_{2}\sum_{j=1}^{m+1}\frac{{\left(e^{-y_{j:k}^{-\beta }}\right)}^{\theta_{2}}-{\left(e^{-y_{j-1:k}^{-\beta }}\right)}^{\theta_{2}}-\phi_{2}\left(m\right)}{\left|{\left(e^{-y_{j:k}^{-\beta }}\right)}^{\theta_{2}}-{\left(e^{-y_{j-1:k}^{-\beta }}\right)}^{\theta_{2}}-\phi_{2}\left(m\right)\right|}\left(\Psi_{j:k}-\Psi_{j-1:k}\right)=0. \end{array}$$

Similarly, the MSALDEs of the unknown parameters *θ*_1_, *θ*_2_, and *β* denoted by ${\hat{\theta }}_{1}^{\text{MSALDE}}$, ${\hat{\theta }}_{2}^{\text{MSALDE}}$, and ${\hat{\beta }}^{\text{MSALDE}}$ can be obtained by minimizing the function that follows:

(21) $$\begin{array}{c} \text{Md}\left(\theta_{1},\theta_{2},\beta \right)=\sum_{i=1}^{n+1}\left|\log\frac{{\left(e^{-x_{i:n}^{-\beta }}\right)}^{\theta_{1}}-{\left(e^{-x_{i-1:n}^{-\beta }}\right)}^{\theta_{1}}}{\phi_{1}\left(n\right)}\right|+\sum_{j=1}^{k+1}\left|\log\frac{{\left(e^{-y_{j:k}^{-\beta }}\right)}^{\theta_{2}}-{\left(e^{-y_{j-1:k}^{-\beta }}\right)}^{\theta_{2}}}{\phi_{2}\left(k\right)}\right|. \end{array}$$

The three following equations are solved:

(22) $$\begin{array}{c} \frac{\partial Md\left(\theta_{1},\theta_{2},\beta \right)}{\partial \theta_{1}}=\sum_{i=1}^{n+1}\frac{\left[\log\left[{\left(e^{-x_{i:n}^{-\beta }}\right)}^{\theta_{1}}-{\left(e^{-x_{i-1:n}^{-\beta }}\right)}^{\theta_{1}}\right]-\log\left(\phi_{1}\left(n\right)\right)\right]}{\left|\log\left[{\left(e^{-x_{i:n}^{-\beta }}\right)}^{\theta_{1}}-{\left(e^{-x_{i-1:n}^{-\beta }}\right)}^{\theta_{1}}\right]-\log\left(\phi_{1}\left(n\right)\right)\right|\left[{\left(e^{-x_{i:n}^{-\beta }}\right)}^{\theta_{1}}-{\left(e^{-x_{i-1:n}^{-\beta }}\right)}^{\theta_{1}}\right]}\\ \times \left[x_{i:n}^{-\beta }{\left(e^{-x_{i:n}^{-\beta }}\right)}^{\theta_{1}}-x_{i-1:n}^{-\beta }{\left(e^{-x_{i-1:n}^{-\beta }}\right)}^{\theta_{1}}\right]=0,\\ \frac{\partial Md\left(\theta_{1},\theta_{2},\beta \right)}{\partial \theta_{2}}=\sum_{j=1}^{k+1}\frac{\left[\log\left[{\left(e^{-y_{j:k}^{-\beta }}\right)}^{\theta_{2}}-{\left(e^{-y_{j-1:k}^{-\beta }}\right)}^{\theta_{2}}\right]-\log\left(\phi_{2}\left(k\right)\right)\right]}{\left|\log\left[{\left(e^{-y_{j:k}^{-\beta }}\right)}^{\theta_{2}}-{\left(e^{-y_{j-1:k}^{-\beta }}\right)}^{\theta_{2}}\right]-\log\left(\phi_{2}\left(k\right)\right)\right|\left[{\left(e^{-y_{j:k}^{-\beta }}\right)}^{\theta_{2}}-{\left(e^{-y_{j-1:k}^{-\beta }}\right)}^{\theta_{2}}\right]}\\ \times \left[y_{j:k}^{-\beta }{\left(e^{-y_{j:k}^{-\beta }}\right)}^{\theta_{2}}-y_{j-1:k}^{-\beta }{\left(e^{-y_{j-1:k}^{-\beta }}\right)}^{\theta_{2}}\right]=0,\\ \frac{\partial Md\left(\theta_{1},\theta_{2},\beta \right)}{\partial \beta }=\theta_{1}\sum_{i=1}^{n+1}\frac{\left[\log\left[{\left(e^{-x_{i:n}^{-\beta }}\right)}^{\theta_{1}}-{\left(e^{-x_{i-1:n}^{-\beta }}\right)}^{\theta_{1}}\right]-\log\left(\phi_{1}\left(n\right)\right)\right]\left(Z_{i:n}-Z_{i-1:n}\right)}{\left|\log\left[{\left(e^{-x_{i:n}^{-\beta }}\right)}^{\theta_{1}}-{\left(e^{-x_{i-1:n}^{-\beta }}\right)}^{\theta_{1}}\right]-\log\left(\phi_{1}\left(n\right)\right)\right|\left[{\left(e^{-x_{i:n}^{-\beta }}\right)}^{\theta_{1}}-{\left(e^{-x_{i-1:n}^{-\beta }}\right)}^{\theta_{1}}\right]}\\ +\theta_{2}\sum_{j=1}^{k+1}\frac{\left[\log\left[{\left(e^{-y_{j:k}^{-\beta }}\right)}^{\theta_{2}}-{\left(e^{-y_{j-1:k}^{-\beta }}\right)}^{\theta_{2}}\right]-\log\left(\phi_{2}\left(k\right)\right)\right]\left(\Psi_{j:k}-\Psi_{j-1:k}\right)}{\left|\log\left[{\left(e^{-y_{j:k}^{-\beta }}\right)}^{\theta_{2}}-{\left(e^{-y_{j-1:k}^{-\beta }}\right)}^{\theta_{2}}\right]-\log\left(\phi_{2}\left(k\right)\right)\right|\left[{\left(e^{-y_{j:k}^{-\beta }}\right)}^{\theta_{2}}-{\left(e^{-y_{j-1:k}^{-\beta }}\right)}^{\theta_{2}}\right]}=0. \end{array}$$

Now, the MSADE and MSALDE of a system *R* can be obtained, respectively:

(23) $$\begin{array}{c} {\hat{R}}^{\text{MSADE}}=\frac{{\hat{\theta }}_{1}^{\text{MSADE}}}{{\hat{\theta }}_{1}^{\text{MSADE}}+{\hat{\theta }}_{2}^{\text{MSADE}}},\\ {\hat{R}}^{\text{MSADE}}=\frac{{\hat{\theta }}_{1}^{\text{MSADE}}}{{\hat{\theta }}_{1}^{\text{MSADE}}+{\hat{\theta }}_{2}^{\text{MSADE}}}. \end{array}$$

### 2.4. Least Square and Weighted Least Square Estimation

Swain et al. [32] proposed the least squares and weighted least squares estimation methods for estimating the Beta distribution parameters. Let *x*_1:*n*_, *x*_2:*n*_,…, *x*_*n*:*n*_ be the order statistics of a random sample of size *n* from EIW (*θ*_1_, *β*) and let *y*_1:*k*_, *y*_2:*k*_,…, *y*_*k*:*k*_ be the order statistics of a random sample of size *k* from EIW (*θ*_2_, *β*). The least square estimations (LEs) of the unknown parameters *θ*_1_, *θ*_2_, and *β* denoted by ${\hat{\theta }}_{1}^{\text{LSE}}$, ${\hat{\theta }}_{2}^{\text{LSE}}$, and ${\hat{\beta }}^{\text{LSE}}$ can be obtained by minimizing the following function with respect to *θ*_1_, *θ*_2_, and *β* as follows:

(24) $$\begin{array}{c} \text{LS}\left(\theta_{1},\theta_{2},\beta \right)=\sum_{i=1}^{n}{\left[F\left(x_{i:n}\right)-\frac{i}{n+1}\right]}^{2}+\sum_{j=1}^{k}{\left[F\left(y_{j:k}\right)-\frac{j}{m+1}\right]}^{2}\\ =\sum_{i=1}^{n}{\left[{\left(e^{-x_{i:n}^{-\beta }}\right)}^{\theta_{1}}-\frac{i}{n+1}\right]}^{2}+\sum_{j=1}^{k}{\left[{\left(e^{-y_{j:k}^{-\beta }}\right)}^{\theta_{2}}-\frac{j}{m+1}\right]}^{2}. \end{array}$$

Instead of minimizing (18), the estimates ${\hat{\theta }}_{1}^{\text{LSE}}$, ${\hat{\theta }}_{2}^{\text{LSE}}$, and ${\hat{\beta }}^{\text{LSE}}$ can be obtained by simultaneously solving the three following equations:

(25) $$\begin{array}{c} \frac{\partial \text{LS}\left(\theta_{1},\theta_{2},\beta \right)}{\partial \theta_{1}}=\sum_{i=1}^{n}x_{i:n}^{-\beta }{\left(e^{-x_{i:n}^{-\beta }}\right)}^{\theta_{1}}\left[{\left(e^{-x_{i:n}^{-\beta }}\right)}^{\theta_{1}}-\frac{i}{n+1}\right]=0,\\ \frac{\partial \text{LS}\left(\theta_{1},\theta_{2},\beta \right)}{\partial \theta_{2}}=\sum_{j=1}^{k}y_{j:k}^{-\beta }{\left(e^{-y_{j:k}^{-\beta }}\right)}^{\theta_{2}}\left[{\left(e^{-y_{j:k}^{-\beta }}\right)}^{\theta_{2}}-\frac{j}{k+1}\right]=0,\\ \frac{\partial \text{LS}\left(\theta_{1},\theta_{2},\beta \right)}{\partial \beta }=\theta_{1}\sum_{i=1}^{n}x_{i:n}^{-\beta }\log\left(x_{i:n}\right){\left(e^{-x_{i:n}^{-\beta }}\right)}^{\theta_{1}}\left[{\left(e^{-x_{i:n}^{-\beta }}\right)}^{\theta_{1}}-\frac{i}{n+1}\right]\\ +\theta_{2}\sum_{j=1}^{k}y_{j:k}\log\left(y_{j:k}\right){\left(e^{-y_{j:k}^{-\beta }}\right)}^{\theta_{2}}{\left[{\left(e^{-y_{j:k}^{-\beta }}\right)}^{\theta_{2}}-\frac{j}{m+1}\right]}^{2}=0. \end{array}$$

Upon obtaining the estimates ${\hat{\theta }}_{1}^{\text{LSE}}$, ${\hat{\theta }}_{2}^{\text{LSE}}$, and ${\hat{\beta }}^{\text{LSE}}$, the LSE of *R* can be obtained as follows:

(26) $$\begin{array}{c} {\hat{R}}^{\text{LSE}}=\frac{{\hat{\theta }}_{1}^{\text{LSE}}}{{\hat{\theta }}_{1}^{\text{LSE}}+{\hat{\theta }}_{2}^{\text{LSE}}}. \end{array}$$

Similarly, the unknown parameters' WLSEs *θ*_1_, *θ*_2_, and *β* denoted by ${\hat{\theta }}_{1}^{\text{WLSE}}$, ${\hat{\theta }}_{2}^{\text{WLSE}}$, and ${\hat{\beta }}^{\text{WLSE}}$ can be obtained by minimizing the following function:

(27) $$\begin{array}{c} \text{WLS}\left(\theta_{1},\theta_{2},\beta \right)=\sum_{i=1}^{n}\omega_{1}\left(i,n\right){\left[{\left(e^{-x_{i:n}^{-\beta }}\right)}^{\theta_{1}}-\frac{i}{n+1}\right]}^{2}+\sum_{j=1}^{k}\omega_{2}\left(j,k\right){\left[{\left(e^{-y_{j:k}^{-\beta }}\right)}^{\theta_{2}}-\frac{j}{m+1}\right]}^{2}, \end{array}$$

where *ω*_1_(*i*, *n*)=((*n*+1)^2^(*n*+2)/*i*(*n* − *i*+1)) and *ω*_2_(*j*, *k*)=((*k*+1)^2^(*k*+2)/*j*(*k* − *j*+1)). These estimates can also be obtained by simultaneously solving the three following equations:

(28) $$\begin{array}{c} \frac{\partial \text{WLS}\left(\theta_{1},\theta_{2},\beta \right)}{\theta_{1}}=\sum_{i=1}^{n}\omega_{1}\left(i,n\right)x_{i:n}^{-\beta }{\left(e^{-x_{i:n}^{-\beta }}\right)}^{\theta_{1}}\left[{\left(e^{-x_{i:n}^{-\beta }}\right)}^{\theta_{1}}-\frac{i}{n+1}\right]=0,\\ \frac{\partial \text{WLS}\left(\theta_{1},\theta_{2},\beta \right)}{\partial \theta_{2}}=\sum_{j=1}^{k}\omega_{2}\left(j,k\right)y_{j:k}^{-\beta }{\left(e^{-y_{j:k}^{-\beta }}\right)}^{\theta_{2}}\left[{\left(e^{-y_{j:k}^{-\beta }}\right)}^{\theta_{2}}-\frac{j}{m+1}\right]=0,\\ \frac{\partial \text{WLS}\left(\theta_{1},\theta_{2},\beta \right)}{\partial \beta }=\theta_{1}\sum_{i=1}^{n}\omega_{1}\left(i,n\right)x_{i:n}^{-\beta }\log\left(x_{i:n}\right){\left(e^{-x_{i:n}^{-\beta }}\right)}^{\theta_{1}}\left[{\left(e^{-x_{i:n}^{-\beta }}\right)}^{\theta_{1}}-\frac{i}{n+1}\right]\\ +\theta_{2}\sum_{j=1}^{k}\omega_{2}\left(j,k\right)y_{j:k}\log\left(y_{j:k}\right){\left(e^{-y_{j:k}^{-\beta }}\right)}^{\theta_{2}}\left[{\left(e^{-y_{j:k}^{-\beta }}\right)}^{\theta_{2}}-\frac{j}{m+1}\right]=0. \end{array}$$

The WLSE of *R* can be obtained as

(29) $$\begin{array}{c} {\hat{R}}^{\text{WLSE}}=\frac{{\hat{\theta }}_{1}^{\text{WLSE}}}{{\hat{\theta }}_{1}^{\text{WLSE}}+{\hat{\theta }}_{2}^{\text{WLSE}}}. \end{array}$$

### 2.5. Cramér-von Mises Estimation

Cramér [33] and von Mises [34] introduced the Cramér-von Mises method of estimation to estimate the unknown parameters *θ*_1_, *θ*_2_, and *β* denoted by ${\hat{\theta }}_{1}^{\text{CME}}$, ${\hat{\theta }}_{2}^{\text{CME}}$, and ${\hat{\beta }}^{\text{CME}}$ which are obtained by minimizing the following goodness-of-fit statistic:

(30) $$\begin{array}{c} \text{CM}\left(\theta_{1},\theta_{2},\beta \right)=\frac{1}{12n}+\frac{1}{12k}+\sum_{i=1}^{n}{\left[{\left(e^{-x_{i:n}^{-\beta }}\right)}^{\theta_{1}}-\varphi_{1}\left(i,n\right)\right]}^{2}+\sum_{j=1}^{k}{\left[{\left(e^{-y_{j:k}^{-\beta }}\right)}^{\theta_{2}}-\varphi_{2}\left(j,k\right)\right]}^{2}, \end{array}$$

with respect to *θ*_1_, *θ*_2_, and *β*, where *φ*_1_(*i*, *n*)=(2(*n* − *i*)+1/2*n*) and *φ*_2_(*j*, *k*)=(2(*k* − *j*)+1/2*k*). These estimates can also be obtained by solving the three following equations simultaneously:

(31) $$\begin{array}{c} \frac{\partial \text{CM}\left(\theta_{1},\theta_{2},\beta \right)}{\partial \theta_{1}}=\sum_{i=1}^{n}x_{i:n}^{-\beta }{\left(e^{-x_{i:n}^{-\beta }}\right)}^{\theta_{1}}\left[{\left(e^{-x_{i:n}^{-\beta }}\right)}^{\theta_{1}}-\varphi_{1}\left(i,n\right)\right]=0,\\ \frac{\partial \text{CM}\left(\theta_{1},\theta_{2},\beta \right)}{\partial \theta_{2}}=\sum_{j-1}^{k}y_{j:k}^{-\beta }{\left(e^{-y_{j:k}^{-\beta }}\right)}^{\theta_{2}}\left[{\left(e^{-y_{j:k}^{-\beta }}\right)}^{\theta_{2}}-\varphi_{2}\left(j,k\right)\right]=0,\\ \frac{\partial \text{CM}\left(\theta_{1},\theta_{2},\beta \right)}{\partial \beta }=\theta_{1}\sum_{i=1}^{n}x_{i:n}^{-\beta }\log\left(x_{i:n}\right){\left(e^{-x_{i:n}^{-\beta }}\right)}^{\theta_{1}}\left[{\left(e^{-x_{i:n}^{-\beta }}\right)}^{\theta_{1}}-\varphi_{1}\left(i,n\right)\right]+\theta_{2}\sum_{j-1}^{k}y_{j:k}^{-\beta }\log\left(y_{j:k}\right)\\ \times {\left(e^{-y_{j:k}^{-\beta }}\right)}^{\theta_{2}}\left[{\left(e^{-y_{j:k}^{-\beta }}\right)}^{\theta_{2}}-\varphi_{2}\left(j,k\right)\right]. \end{array}$$

The CME of *R* can be obtained as follows:

(32) $$\begin{array}{c} {\hat{R}}^{\text{CME}}=\frac{{\hat{\theta }}_{1}^{\text{CME}}}{{\hat{\theta }}_{1}^{\text{CME}}+{\hat{\theta }}_{2}^{\text{CME}}}. \end{array}$$

### 2.6. Anderson-Darling Estimation

Another type of minimum distance estimator is the Anderson-Darling estimation, which is obtained by minimizing Anderson-Darling statistics. Right-tail Anderson-Darling estimation (ADEs) statistics were introduced by Luceño [35] as a modification to the Anderson-Darling estimation (ADEs) statistics (RADEs). The unknown parameters of ADEs *θ*_1_, *θ*_2_, and *β* denoted by ${\hat{\theta }}_{1}^{\text{ADE}}$, ${\hat{\theta }}_{2}^{\text{ADE}}$, and ${\hat{\beta }}^{\text{ADE}}$ are obtained by minimizing the following function:

(33) $$\begin{array}{c} \text{ADE}\left(\theta_{1},\theta_{2},\beta \right)=-n-k-\frac{1}{n}\sum_{i=1}^{n}\left(2i-1\right)\left[\log\left(e^{-\theta_{1}x_{i:n}^{-\beta }}\right)-\theta_{1}x_{n+1-i:n}^{-\beta }\right]-\frac{1}{k}\sum_{j=1}^{k}\left(2j-1\right)\left[\log\left(e^{-\theta_{2}y_{j:k}^{-\beta }}\right)-\theta_{2}y_{k+1-j:k}^{-\beta }\right], \end{array}$$

with respect to *θ*_1_, *θ*_2_, and *β*. These estimates can also be obtained by solving the three following equations simultaneously:

(34) $$\begin{array}{c} \frac{\partial \text{ADE}\left(\theta_{1},\theta_{2},\beta \right)}{\partial \theta_{1}}=\frac{1}{n}\sum_{i=1}^{n}\left(2i-1\right)\left[\left(\frac{x_{i:n}^{-\beta }e^{-\theta_{1}x_{i:n}^{-\beta }}}{e^{-\theta_{1}x_{i:n}^{-\beta }}}\right)+x_{n+1-i:n}^{-\beta }\right]=0,\\ \frac{\partial \text{ADE}\left(\theta_{1},\theta_{2},\beta \right)}{\partial \theta_{2}}=\frac{1}{k}\sum_{j=1}^{k}\left(2j-1\right)\left[\left(\frac{y_{j:k}^{-\beta }e^{-\theta_{2}y_{j:k}^{-\beta }}}{e^{-\theta_{2}y_{j:k}^{-\beta }}}\right)+y_{k+1-j:k}^{-\beta }\right]=0,\\ \frac{\partial \text{ADE}\left(\theta_{1},\theta_{2},\beta \right)}{\partial \beta }=\frac{\theta_{1}}{n}\sum_{i=1}^{n}\left(2i-1\right)\left[\frac{x_{i:n}^{-\beta }\log\left(x_{i:n}\right)e^{-\theta_{1}x_{i:n}^{-\beta }}}{e^{-\theta_{1}x_{i:n}^{-\beta }}}-x_{n+1-i:n}^{-\beta }\log\left(x_{n+1-i:n}\right)\right]\\ +\frac{\theta_{2}}{k}\sum_{j=1}^{k}\left(2j-1\right)\left[\left(\frac{y_{j:k}^{-\beta }\log\left(y_{j:k}\right)e^{-\theta_{2}y_{j:k}^{-\beta }}}{e^{-\theta_{2}y_{j:k}^{-\beta }}}\right)-y_{k+1-j:k}^{-\beta }\log\left(y_{k+1-j:k}\right)\right]=0. \end{array}$$

The ADE of *R* can be obtained, respectively, by

(35) $$\begin{array}{c} {\hat{R}}^{\text{ADE}}=\frac{{\hat{\theta }}_{1}^{\text{ADE}}}{{\hat{\theta }}_{1}^{\text{ADE}}+{\hat{\theta }}_{2}^{\text{ADE}}}. \end{array}$$

## 3. Bootstrap Confidence Intervals

There are two confidence intervals for parameters *θ*_1_, *θ*_2_, and *β* in this section, and parametric bootstrap methods will be proposed. Percentile bootstrap (Boot-P) and bias-corrected percentile bootstrap (Boot-BCP) confidence intervals are shown as two distinct parametric confidence intervals. The steps below will show how to estimate the confidence intervals of *R*.

### 3.1. Boot-P Confidence

1. Generate samples (*x*_1:*n*_, *x*_2:*n*_,…, *x*_*n*:*n*_) and (*y*_*i*:*k*_, *y*_2:*k*_,…, *y*_*k*:*k*_) to obtain the bootstrap estimates of ${\hat{\theta }}_{1}^{*},{\hat{\theta }}_{2}^{*},{\hat{\beta }}^{*}$, and ${\hat{R}}^{*}$ from the original data, where ${\hat{\theta }}_{1}^{*},{\hat{\theta }}_{2}^{*},{\hat{\beta }}^{*}$, and ${\hat{R}}^{*}$ are the estimates obtained from the different estimation.
2. Use ${\hat{\theta }}_{1}^{*}$ and ${\hat{\beta }}^{*}$ to generate a bootstrap sample (*x*_1:*n*_^Boot^, *x*_2:*n*_^Boot^,…, *x*_*n*:*n*_^Boot^) and ${\hat{\theta }}_{2}^{*}$ and ${\hat{\beta }}^{*}$ to generate a bootstrap sample (*y*_1:*k*_^Boot^, *y*_2:*k*_^Boot^,…, *y*_*k*:*k*_^Boot^).
3. Based on (*x*_1:*n*_^Boot^, *x*_2:*n*_^Boot^,…, *x*_*n*:*n*_^Boot^) and (*y*_1:*k*_^Boot^, *y*_2:*k*_^Boot^,…, *y*_*k*:*k*_^Boot^), obtain the bootstrap estimate of a system *R*, say ${\hat{R}}^{\text{Boot}}$.
4. Repeat steps 1–3 *B* times to have $\left({\hat{R}}^{\text{Boot}\left(1\right)},{\hat{R}}^{\text{Boot}\left(2\right)},\ldots ,{\hat{R}}^{\text{Boot}\left(B\right)}\right)$.
5. Arrange the bootstrap estimates in step 4 in ascending order as $\left({\hat{R}}^{\text{Boot}\left[1\right]},{\hat{R}}^{\text{Boot}\left[2\right]},\ldots ,{\hat{R}}^{\text{Boot}\left[B\right]}\right)$.
6. A two-side 100(1 − *γ*)% Boot-P confidence interval of *R* is given by $\left\{{\hat{R}}^{\text{Boot}\left[B\left(\gamma /2\right)\right]},{\hat{R}}^{\text{Boot}\left[B\left(1-\gamma /2\right)\right]}\right\}$.

### 3.2. Boot-BCP Confidence Interval

(1) The same steps as (1–4) in Boot-P
(2) A two-side 100(1 − *γ*)% Boot-BCP confidence interval for the unknown parameters is given by
  (36) $$\begin{array}{c} \left\{{\hat{R}}^{\text{Boot}\left[B\delta_{1}\right]},{\hat{R}}^{\text{Boot}\left[B\delta_{2}\right]}\right\}, \end{array}$$
where
  (37) $$\begin{array}{c} \delta_{1}=\Phi \left(2z_{0}+z_{\left(\gamma /2\right)}\right),\\ \delta_{2}=\Phi \left(2z_{0}+z_{\left(1-\gamma /2\right)}\right), \end{array}$$
where Φ(.) is the CDF of the standard normal distribution, *z*_*γ*_=Φ^−1^(*γ*), and *z*_0_ can be obtained as follows:
  (38) $$\begin{array}{c} z_{0}=\Phi^{-1}\left(\frac{{\hat{R}}^{\text{Boot}\left[i\right]}}{B}\right),\;i=1,2,\ldots ,B. \end{array}$$

## 4. Simulation Study

In the simulation section, a Monte Carlo simulation is done to estimate the unknown parameters of EIW distribution to get stress-strength reliability for MLE, MPSE, MSADE, MSALDE, LSE, WLSE, CME, and ADE methods using R-program are described as follows:

Step 1: Generate 10000 random samples, in strength variable (*X*), the sample size is *n*= 30,  35,  50 , and 70 from the EIW distribution, and in stress variable the sample size is *m*= 40,  45,  60 , and  80 from the EIW distribution.
Step 2: Use the quantile **x**_**i**_=(−[ln(*q*_*i*_)]^1/*θ*_1_^)^−1/*β*^, **y**_**i**_=(−[ln(*q*_*i*_)]^1/*θ*_2_^)^−1/*β*^; 0 < *q*_*i*_ < 1, where *x* and *y* are distributed as EIW for different parameters (*θ*_1_, *θ*_2_, *β*) and different cases of actual parameters values are selected; see Tables 1–3.
    Case 1: *θ*_1_=0.1 and 3, *θ*_2_=0.1, and *β*=0.5.
    Case 2: *θ*_2_=0.4 and 2, *θ*_1_=3, and *β*=0.9.
    Case 3: *θ*_2_=0.4 and 1.5, *θ*_1_=0.5, and *β*=0.5.
Step 3: The MLE, MPSE, MSADE, MSALDE, LSE, WLSE, CME, and ADE of the model parameters are obtained by solving the nonlinear equations for the stress-strength model.
Step 4: The mean and mean square errors (MSE) of the parameters are obtained.
Step 5: The length of CI by using bootstrapping of the stress-strength reliability is obtained in Tables 4–6.
Step 6: The numerical results of parameters estimation of EIW distribution are listed in Tables 1–3.

The simulation outcomes of point estimation are recorded in Tables 1–3. The following concluding remakes are noticed based on these tables:

- In some cases, as the sample size of strength increases and for a fixed sample size of stress, the MSEs associated with the parameter estimates decrease for all methods of estimation.
- In some cases, as the sample size of stress increases and for a fixed sample size of strength, the MSEs associated with the parameter estimates decrease for all methods of estimation.
- In Case 1, as *θ*_1_ increases and for a fixed sample size of strength and stress, the MSEs of parameters of EIW are increasing for all methods of estimation.
- In Case 2, as *θ*_2_ increases and for a fixed sample size of strength and stress, the MSEs for most of the parameters of EIW are increasing for all methods of estimation.

The simulation outcomes of interval estimation of stress-strength reliability are recorded in Tables 4–6. The following concluding remakes are noticed based on these tables:

- In some cases, as the sample size of strength increases and for a fixed sample size of stress, the length of CI of stress-strength reliability estimates decreases for all methods of estimation.
- In some cases, as the sample size of stress increases and for a fixed sample size of strength, the length of CI of stress-strength reliability estimates decreases for all methods of estimation.
- In some cases, as a level of interval increases and for a fixed sample size of stress and strength, the length of CI of stress-strength reliability estimates increases for all methods of estimation.

## 5. Application of Real Data

In this section, we consider two applications of the stress-strength reliability model by using breaking strengths of jute fiber and carbon fibers data to describe all the details for illustrative purposes. We used Kolmogorov-Smirnov statistics (KSS) with *P* value to check the fit of the model and standard errors (SE) of estimators.

### 5.1. Breaking Strengths of Jute Fiber Data

A pair of real data sets are studied for demonstration purposes. The breaking strengths of jute fiber at two different gauge lengths are depicted in these data. Xia et al. [36] used these two data sets in their study, where *X* represents the breaking strength of jute fiber with a diameter of 10 mm and *Y* represents the breaking strength of a 20 mm diameter jute fiber.

The breaking strengths of jute fiber with a gauge length of 10 mm are “*X* = 693.73, 704.66, 323.83, 778.17, 123.06, 637.66, 383.43, 151.48, 108.94, 50.16, 671.49, 183.16, 257.44, 727.23, 291.27, 101.15, 376.42, 163.40, 141.38, 700.74, 262.90, 353.24, 422.11, 43.93, 590.48, 212.13, 303.90, 506.60, 530.55, and 177.25.”

The breaking strengths of jute fibers with a gauge length of 20 mm are “*Y* = 71.46, 419.02, 284.64, 585.57, 456.60, 113.85, 187.85, 688.16, 662.66, 45.58, 578.62, 756.70, 594.29, 166.49, 99.72, 707.36, 765.14, 187.13, 145.96, 350.70, 547.44, 116.99, 375.81, 581.60, 119.86, 48.01, 200.16, 36.75, 244.53, and 83.55.”

**Figure 2:**
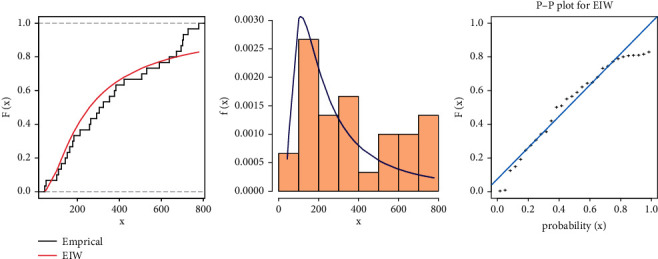
Cumulative function and empirical CDF, histogram, and P-P plot for the EIW distribution for *X* of breaking strengths of jute fiber data.

**Figure 3:**
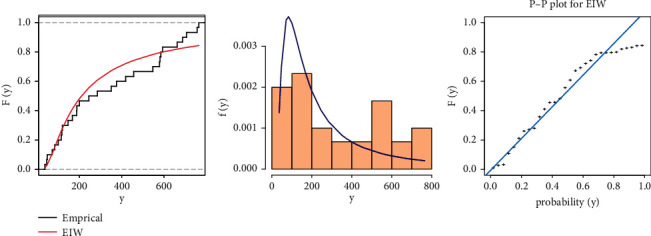
Cumulative function and empirical CDF, histogram, and P-P plot for the EIW distribution for *Y* of breaking strengths of jute fiber data.

From Table 7, we can see that although the EIW distribution fits the data because the diﬀerence between the values of KSS is very small and the *P* value is more than 0.05, for more illustration, Figures 2 and 3 show the fitted CDF with empirical CDF, fitted PDF with histogram, and P-P plot for strength and stress, respectively, computed at the estimated parameters of EIW distribution.

The estimates of the parameters model of stress-strength reliability for EIW distribution are obtained in Table 8. MSADE has the smallest SE and the largest reliability.

### 5.2. Carbon Fibers Data

In this subsection, we look at two data sets and discuss all of the specifics for the sake of illustration. The two data sets were first published by Bader and Priest [37]; and they reflected the GPA strength of single carbon fibers with lengths of 10 mm (Data Set I) and 10 mm (Data Set II), respectively, with sample sizes of *n* = 63 and *m* = 69. These data were analyzed previously by Hassan et al. [38]. The following are the data sets:

  Data Set I (length of 10 mm): *X* (*n* = 63): “1.901, 2.132, 2.203, 2.228, 2.257, 2.350, 2.361, 2.396, 2.397, 2.445, 2.454, 2.474, 2.518, 2.522, 2.525, 2.532, 2.575, 2.614, 2.616, 2.618, 2.624, 2.659, 2.675, 2.738, 2.740, 2.856, 2.917, 2.928, 2.937, 2.937, 2.977, 2.996, 3.030, 3.125, 3.139, 3.145, 3.220, 3.223, 3.235, 3.243, 3.264, 3.272, 3.294, 3.332, 3.346, 3.377, 3.408, 3.435, 3.493, 3.501, 3.537, 3.554, 3.562, 3.628, 3.852, 3.871, 3.886, 3.971, 4.024, 4.027, 4.225, 4.395, 5.020.”
  Data Set II (length of 20 mm): *Y* (*m* = 69): “1.312, 1.314, 1.479, 1.552, 1.700, 1.803, 1.861, 1.865, 1.944, 1.958, 1.966, 1.997, 2.006, 2.021, 2.027, 2.055, 2.063, 2.098, 2.14, 2.179, 2.224, 2.240, 2.253, 2.270, 2.272, 2.274, 2.301, 2.301, 2.359, 2.382, 2.382, 2.426, 2.434, 2.435, 2.478, 2.490, 2.511, 2.514, 2.535, 2.554, 2.566, 2.57, 2.586, 2.629, 2.633, 2.642, 2.648, 2.684, 2.697, 2.726, 2.770, 2.773, 2.800, 2.809, 2.818, 2.821, 2.848, 2.88, 2.954, 3.012, 3.067, 3.084, 3.090, 3.096, 3.128, 3.233, 3.433, 3.585, 3.585.”

**Figure 4:**
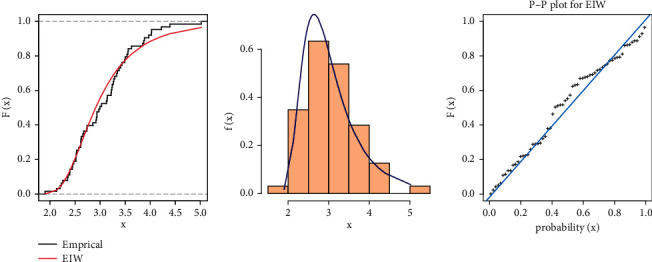
Cumulative function and empirical CDF, histogram, and P-P plot for the EIW distribution for Data Set I of carbon fibers data.

**Figure 5:**
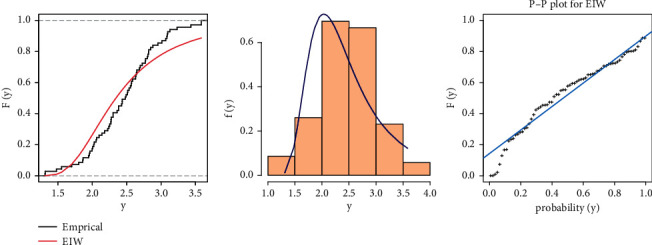
Cumulative function and empirical CDF, histogram, and P-P plot for the EIW distribution for Data Set II of carbon fibers data.

From Table 9, we can see that although the EIW distribution fits the carbon fibers data because the diﬀerence between the values of KSS is very small and the *P* value is more than 0.05, for more illustration, Figures 4 and 5 show the fitted CDF with empirical CDF, fitted PDF with histogram, and P-P plot for strength and stress, respectively, computed at the estimated parameters of EIW distribution.

The estimates of the parameters model of stress-strength reliability for EIW distribution are obtained in Table 10. MSADE has the smallest SE and the largest reliability.

## 6. Conclusion

In this paper, we assumed that *X* and *Y* are two independent EIW distributions with the same scale parameter, and by using eight methods of estimation, we could propose the estimations of the unknown parameters *θ*_1_, *θ*_2_, and *β* and the system of stress-strength parameter *R*=*P*(*Y* < *X*). The eight methods of estimations are MLEs, MPSEs, MSADEs, MSALDEs, LSEs, WLSEs, CMEs, and ADEs. The percentile bootstrap and bias-corrected percentile bootstrap confidence intervals which are two parametric bootstrap confidence intervals of *R* were introduced. Breaking strengths of jute fiber and carbon fibers data were used as two real data sets to demonstrate the performance of the unknown parameters *θ*_1_, *θ*_2_, and *β* and the system of stress-strength reliability *R*=*P*(*Y* < *X*) in practical applications, the goodness of fit of the methods estimators for each real data set was examined using the KSS, and the results were sufficient and satisfactory.

We investigated the proposed point and interval estimates using simulation studies, and they performed admirably for a variety of sample sizes, as evidenced by their MSE and confidence intervals. In both techniques, the MSE decreases as the sample size increases; however, the method of maximum product of spacing outperforms other estimation methods. By comparing the estimators using an extensive Monte Carlo numerical simulation study and analyzing a real-world data set, in all sample cases, the MPSEs method outperformed the MLEs. Overall, simulation results show that the maximum product of spacing methods outperforms the other methods in terms of minimum MSE and confidence interval length in the majority of cases. In terms of minimum confidence interval lengths, Boot‐PCP outperforms Boot‐P confidence intervals.

## Figures and Tables

**Table 1:** Mean and MSE for parameter and stress-strength reliability for EIW distribution with different sample size: Case 1.

*θ*_2_=0.1, *β*=0.5			*n* = 30, *m* = 35				*n* = 40, *m* = 45				*n* = 30, *m* = 35				*n* = 40, *m* = 45			
*θ*_1_			*θ*_1_	*θ*_2_	*β*	*R*	*θ*_1_	*θ*_2_	*β*	*R*	*θ*_1_	*θ*_2_	*β*	*R*	*θ*_1_	*θ*_2_	*β*	*R*
0.1	MLE	Mean	0.0987	0.0981	0.5147	0.5003	0.1002	0.0984	0.5135	0.5039	0.0994	0.0986	0.5096	0.5021	0.0991	0.0994	0.5064	0.4993
		MSE	0.0010	0.0009	0.0026	0.0036	0.0009	0.0008	0.0023	0.0033	0.0006	0.0006	0.0017	0.0023	0.0004	0.0004	0.0011	0.0016
	MPSE	Mean	0.0926	0.0937	0.5098	0.4958	0.0952	0.0948	0.5084	0.5003	0.0961	0.0961	0.5049	0.5002	0.0968	0.0975	0.5024	0.4983
		MSE	0.0010	0.0009	0.0025	0.0036	0.0008	0.0008	0.0023	0.0033	0.0006	0.0006	0.0016	0.0023	0.0004	0.0004	0.0011	0.0016
	MSADE	Mean	0.1792	0.1796	0.4323	0.4977	0.1722	0.1688	0.4365	0.5035	0.1430	0.1410	0.4541	0.5020	0.1265	0.1266	0.4670	0.4990
		MSE	0.0237	0.0237	0.0127	0.0047	0.0184	0.0168	0.0109	0.0046	0.0068	0.0060	0.0063	0.0032	0.0027	0.0026	0.0038	0.0025
	MSALDE	Mean	0.1104	0.1120	0.4854	0.4950	0.1112	0.1105	0.4866	0.5005	0.1086	0.1086	0.4874	0.4997	0.1067	0.1076	0.4881	0.4976
		MSE	0.0018	0.0017	0.0034	0.0044	0.0016	0.0014	0.0031	0.0040	0.0011	0.0011	0.0022	0.0030	0.0007	0.0007	0.0015	0.0021
	LSE	Mean	0.1094	0.1087	0.4907	0.4998	0.1096	0.1079	0.4933	0.5038	0.1064	0.1053	0.4951	0.5023	0.1036	0.1041	0.4967	0.4987
		MSE	0.0014	0.0013	0.0031	0.0044	0.0013	0.0012	0.0030	0.0041	0.0008	0.0008	0.0022	0.0029	0.0006	0.0006	0.0016	0.0021
	WLSE	Mean	0.1050	0.1045	0.5001	0.5000	0.1054	0.1038	0.5022	0.5038	0.1028	0.1019	0.5026	0.5023	0.1009	0.1014	0.5026	0.4987
		MSE	0.0012	0.0011	0.0027	0.0042	0.0011	0.0010	0.0027	0.0039	0.0007	0.0007	0.0020	0.0027	0.0005	0.0005	0.0014	0.0019
	CME	Mean	0.1009	0.1000	0.5126	0.5002	0.1021	0.1003	0.5126	0.5042	0.1009	0.0998	0.5089	0.5025	0.0996	0.1001	0.5068	0.4987
		MSE	0.0013	0.0011	0.0036	0.0048	0.0011	0.0011	0.0034	0.0045	0.0008	0.0007	0.0025	0.0031	0.0005	0.0005	0.0017	0.0022
	ADE	Mean	0.1299	0.1408	0.4502	0.4794	0.1407	0.1511	0.4338	0.4828	0.1527	0.1638	0.4102	0.4829	0.1565	0.1683	0.4011	0.4824
		MSE	0.0024	0.0033	0.0047	0.0038	0.0031	0.0043	0.0064	0.0032	0.0038	0.0054	0.0094	0.0021	0.0040	0.0056	0.0108	0.0016

3	MLE	Mean	3.2110	0.0973	0.5160	0.9689	3.1597	0.0980	0.5144	0.9686	3.1236	0.0981	0.5129	0.9685	3.0789	0.0993	0.5069	0.9681
		MSE	0.5946	0.0008	0.0026	0.0001	0.3823	0.0008	0.0023	0.0001	0.2778	0.0006	0.0017	0.0001	0.1584	0.0004	0.0012	0.0001
	MPSE	Mean	2.9156	0.0927	0.5115	0.9674	2.8999	0.0942	0.5097	0.9672	2.9247	0.0955	0.5083	0.9673	2.9281	0.0974	0.5029	0.9671
		MSE	0.4548	0.0008	0.0025	0.0002	0.3102	0.0007	0.0023	0.0001	0.2356	0.0006	0.0016	0.0001	0.1407	0.0004	0.0011	0.0001
	MSADE	Mean	2.9170	0.1824	0.4295	0.9393	2.9025	0.1726	0.4353	0.9422	2.9180	0.1459	0.4549	0.9507	2.9214	0.1283	0.4660	0.9568
		MSE	0.4310	0.0236	0.0138	0.0025	0.3653	0.0187	0.0117	0.0020	0.2840	0.0095	0.0072	0.0011	0.2035	0.0034	0.0041	0.0004
	MSALDE	Mean	2.9198	0.1111	0.4865	0.9612	2.8972	0.1097	0.4880	0.9618	2.9226	0.1085	0.4910	0.9628	2.9213	0.1070	0.4896	0.9638
		MSE	0.4766	0.0016	0.0033	0.0003	0.3437	0.0015	0.0031	0.0003	0.2801	0.0011	0.0023	0.0002	0.1748	0.0007	0.0016	0.0001
	LSE	Mean	3.0243	0.1085	0.4920	0.9625	3.0018	0.1073	0.4942	0.9634	3.0158	0.1053	0.4977	0.9645	2.9985	0.1044	0.4955	0.9652
		MSE	0.6812	0.0013	0.0035	0.0003	0.4386	0.0011	0.0029	0.0002	0.3524	0.0009	0.0023	0.0002	0.2180	0.0006	0.0016	0.0001
	WLSE	Mean	3.1026	0.1042	0.5015	0.9649	3.0730	0.1033	0.5031	0.9656	3.0683	0.1020	0.5047	0.9664	3.0458	0.1016	0.5017	0.9668
		MSE	0.6865	0.0011	0.0032	0.0002	0.4490	0.0009	0.0027	0.0002	0.3410	0.0007	0.0020	0.0001	0.2010	0.0005	0.0014	0.0001
	CME	Mean	3.2307	0.0998	0.5140	0.9672	3.1767	0.0997	0.5135	0.9676	3.1399	0.0998	0.5116	0.9675	3.0866	0.1004	0.5056	0.9674
		MSE	0.9303	0.0011	0.0040	0.0002	0.5731	0.0010	0.0034	0.0002	0.4296	0.0008	0.0026	0.0002	0.2502	0.0005	0.0017	0.0001
	ADE	Mean	2.7352	0.1403	0.4516	0.9484	2.5983	0.1504	0.4348	0.9432	2.4530	0.1635	0.4128	0.9357	2.3875	0.1681	0.4012	0.9329
		MSE	0.4464	0.0034	0.0049	0.0008	0.3747	0.0041	0.0062	0.0010	0.4385	0.0054	0.0090	0.0014	0.4626	0.0056	0.0108	0.0015

**Table 2:** Mean and MSE for parameter and stress-strength reliability for EIW distribution with different sample size: Case 2.

*θ*_1_=3, *β*=0.9			*n*=30, *m*=40				*n*=35, *m*=45				*n*=50, *m*=60				*n*=70, *m*=80			
*θ*_2_			*θ*_1_	*θ*_2_	*β*	*R*	*θ*_1_	*θ*_2_	*β*	*R*	*θ*_1_	*θ*_2_	*β*	*R*	*θ*_1_	*θ*_2_	*β*	*R*
0.4	MLE	Mean	3.2053	0.4014	0.9253	0.8851	3.1786	0.3953	0.9287	0.8862	3.1287	0.4009	0.9197	0.8842	3.0716	0.4026	0.9133	0.8826
		MSE	0.4749	0.0068	0.0086	0.0010	0.4206	0.0059	0.0076	0.0009	0.2637	0.0041	0.0053	0.0006	0.1724	0.0036	0.0040	0.0005
	MPSE	Mean	2.9079	0.3779	0.9167	0.8813	2.9143	0.3749	0.9200	0.8828	2.9277	0.3858	0.9107	0.8813	2.9203	0.3909	0.9057	0.8804
		MSE	0.3645	0.0064	0.0084	0.0010	0.3292	0.0059	0.0073	0.0009	0.2188	0.0040	0.0050	0.0006	0.1552	0.0034	0.0038	0.0005
	MSADE	Mean	2.9214	0.5592	0.7587	0.8375	2.9088	0.5181	0.7894	0.8463	2.9256	0.4886	0.8104	0.8545	2.9095	0.4515	0.8424	0.8635
		MSE	0.3840	0.0847	0.0532	0.0060	0.3938	0.0592	0.0412	0.0047	0.2788	0.0330	0.0269	0.0030	0.2062	0.0123	0.0129	0.0015
	MSALDE	Mean	2.9212	0.4160	0.8712	0.8707	2.9121	0.4073	0.8824	0.8733	2.9187	0.4129	0.8797	0.8729	2.9308	0.4113	0.8828	0.8749
		MSE	0.4403	0.0101	0.0116	0.0016	0.3869	0.0085	0.0103	0.0013	0.2825	0.0063	0.0073	0.0011	0.1981	0.0051	0.0054	0.0008
	LSE	Mean	3.0273	0.4158	0.8858	0.8741	3.0334	0.4078	0.8924	0.8766	3.0265	0.4088	0.8933	0.8777	3.0005	0.4082	0.8953	0.8779
		MSE	0.5115	0.0079	0.0115	0.0014	0.5047	0.0067	0.0093	0.0013	0.3067	0.0050	0.0072	0.0009	0.2222	0.0042	0.0053	0.0007
	WLSE	Mean	3.0998	0.4103	0.9021	0.8782	3.0922	0.4028	0.9071	0.8803	3.0786	0.4047	0.9062	0.8810	3.0396	0.4049	0.9051	0.8805
		MSE	0.5286	0.0075	0.0102	0.0013	0.4903	0.0064	0.0084	0.0011	0.2912	0.0047	0.0061	0.0008	0.2046	0.0039	0.0046	0.0006
	CME	Mean	3.2332	0.4054	0.9255	0.8831	3.2120	0.3986	0.9272	0.8844	3.1515	0.4022	0.9183	0.8834	3.0891	0.4033	0.9135	0.8821
		MSE	0.7092	0.0080	0.0133	0.0013	0.6686	0.0069	0.0109	0.0012	0.3789	0.0050	0.0080	0.0009	0.2562	0.0042	0.0058	0.0006
	ADE	Mean	2.7429	0.4821	0.8131	0.8462	2.6222	0.4879	0.7865	0.8392	2.4637	0.5053	0.7417	0.8274	2.3850	0.5079	0.7239	0.8227
		MSE	0.3628	0.0153	0.0157	0.0029	0.3866	0.0151	0.0194	0.0033	0.4131	0.0162	0.0296	0.0041	0.4649	0.0157	0.0344	0.0044

2	MLE	Mean	3.2020	2.0834	0.9255	0.6023	3.1916	2.0616	0.9265	0.6045	3.1297	2.0609	0.9160	0.6010	3.0899	2.0468	0.9129	0.6008
		MSE	0.5430	0.1455	0.0086	0.0038	0.4391	0.1066	0.0072	0.0032	0.3000	0.0888	0.0050	0.0023	0.1536	0.0635	0.0038	0.0016
	MPSE	Mean	2.9078	1.9305	0.9170	0.5974	2.9284	1.9252	0.9185	0.6004	2.9313	1.9522	0.9080	0.5983	2.9375	1.9607	0.9055	0.5990
		MSE	0.4307	0.1208	0.0084	0.0038	0.3388	0.0944	0.0070	0.0031	0.2509	0.0770	0.0048	0.0023	0.1331	0.0568	0.0037	0.0016
	MSADE	Mean	2.9405	2.0430	0.7548	0.5852	2.9342	2.0228	0.7748	0.5890	2.9056	2.0005	0.8134	0.5902	2.9083	2.0042	0.8375	0.5908
		MSE	0.5834	0.1433	0.0581	0.0052	0.3819	0.1279	0.0458	0.0042	0.2873	0.0998	0.0249	0.0032	0.2127	0.0859	0.0133	0.0026
	MSALDE	Mean	2.9186	1.9557	0.8715	0.5945	2.9174	1.9440	0.8805	0.5965	2.9185	1.9645	0.8781	0.5952	2.9253	1.9774	0.8800	0.5956
		MSE	0.5077	0.1354	0.0114	0.0046	0.4118	0.1127	0.0090	0.0039	0.2998	0.0888	0.0070	0.0028	0.1693	0.0699	0.0051	0.0021
	LSE	Mean	3.0229	2.0093	0.8855	0.5962	3.0341	2.0046	0.8929	0.5983	3.0183	2.0074	0.8899	0.5985	3.0130	2.0181	0.8947	0.5975
		MSE	0.5768	0.1531	0.0107	0.0045	0.4872	0.1232	0.0088	0.0038	0.3191	0.1065	0.0066	0.0027	0.2120	0.0788	0.0051	0.0021
	WLSE	Mean	3.0951	2.0375	0.9017	0.5984	3.0977	2.0276	0.9068	0.6005	3.0720	2.0313	0.9024	0.5998	3.0549	2.0351	0.9048	0.5990
		MSE	0.5913	0.1455	0.0096	0.0044	0.4918	0.1121	0.0080	0.0036	0.3177	0.0979	0.0057	0.0026	0.1933	0.0719	0.0044	0.0019
	CME	Mean	3.2290	2.1025	0.9251	0.6006	3.2123	2.0851	0.9277	0.6022	3.1428	2.0647	0.9148	0.6012	3.1019	2.0600	0.9130	0.5995
		MSE	0.7932	0.1973	0.0123	0.0049	0.6467	0.1538	0.0104	0.0040	0.3902	0.1248	0.0071	0.0029	0.2465	0.0895	0.0055	0.0021
	ADE	Mean	2.7363	2.0452	0.8123	0.5692	2.6301	1.9800	0.7864	0.5679	2.4633	1.8918	0.7389	0.5642	2.3933	1.8481	0.7234	0.5635
		MSE	0.4053	0.1175	0.0156	0.0047	0.3877	0.0785	0.0192	0.0040	0.4310	0.0675	0.0303	0.0032	0.4488	0.0604	0.0342	0.0027

**Table 3:** Mean and MSE for parameter and stress-strength reliability for EIW distribution with different sample size: Case 3.

*θ*_1_=0.5, *β*=0.5			*n*=30, *m*=40				*n*=35, *m*=45				*n*=50, *m*=60				*n*=70, *m*=80			
*θ*_2_			*θ*_1_	*θ*_2_	*β*	*R*	*θ*_1_	*θ*_2_	*β*	*R*	*θ*_1_	*θ*_2_	*β*	*R*	*θ*_1_	*θ*_2_	*β*	*R*
0.4	MLE	Mean	0.5086	0.4038	0.5127	0.5560	0.5054	0.4008	0.5136	0.5565	0.5051	0.3997	0.5081	0.5577	0.5019	0.3996	0.5058	0.5564
		MSE	0.0120	0.0067	0.0026	0.0035	0.0108	0.0062	0.0024	0.0035	0.0072	0.0046	0.0016	0.0025	0.0049	0.0032	0.0010	0.0017
	MPSE	Mean	0.4690	0.3800	0.5082	0.5512	0.4715	0.3801	0.5087	0.5526	0.4805	0.3839	0.5039	0.5554	0.4843	0.3881	0.5017	0.5548
		MSE	0.0110	0.0063	0.0026	0.0035	0.0099	0.0059	0.0023	0.0034	0.0068	0.0045	0.0016	0.0025	0.0047	0.0031	0.0010	0.0017
	MSADE	Mean	0.6571	0.5486	0.4237	0.5455	0.6203	0.5088	0.4372	0.5493	0.5813	0.4714	0.4532	0.5519	0.5501	0.4475	0.4659	0.5514
		MSE	0.0838	0.0698	0.0149	0.0045	0.0552	0.0427	0.0107	0.0044	0.0318	0.0221	0.0071	0.0031	0.0150	0.0115	0.0040	0.0024
	MSALDE	Mean	0.5142	0.4220	0.4825	0.5481	0.5138	0.4149	0.4860	0.5518	0.5113	0.4109	0.4870	0.5540	0.5060	0.4068	0.4893	0.5540
		MSE	0.0149	0.0099	0.0035	0.0044	0.0143	0.0087	0.0030	0.0042	0.0094	0.0065	0.0024	0.0029	0.0065	0.0044	0.0014	0.0022
	LSE	Mean	0.5200	0.4171	0.4899	0.5527	0.5142	0.4111	0.4946	0.5545	0.5114	0.4081	0.4926	0.5554	0.5086	0.4055	0.4948	0.5558
		MSE	0.0145	0.0075	0.0034	0.0043	0.0120	0.0070	0.0029	0.0041	0.0081	0.0051	0.0022	0.0028	0.0058	0.0035	0.0015	0.0021
	WLSE	Mean	0.5150	0.4118	0.4991	0.5536	0.5103	0.4068	0.5027	0.5552	0.5077	0.4041	0.5000	0.5562	0.5054	0.4019	0.5008	0.5565
		MSE	0.0138	0.0072	0.0030	0.0041	0.0116	0.0067	0.0027	0.0039	0.0077	0.0049	0.0019	0.0027	0.0054	0.0034	0.0013	0.0020
	CME	Mean	0.5131	0.4068	0.5118	0.5553	0.5078	0.4019	0.5140	0.5568	0.5065	0.4015	0.5064	0.5571	0.5050	0.4006	0.5049	0.5570
		MSE	0.0153	0.0076	0.0038	0.0047	0.0126	0.0071	0.0033	0.0044	0.0084	0.0051	0.0023	0.0030	0.0059	0.0036	0.0016	0.0022
	ADE	Mean	0.5442	0.4842	0.4498	0.5281	0.5504	0.4914	0.4359	0.5280	0.5643	0.5056	0.4082	0.5274	0.5670	0.5045	0.4009	0.5292
		MSE	0.0139	0.0153	0.0049	0.0042	0.0122	0.0159	0.0061	0.0038	0.0104	0.0165	0.0098	0.0027	0.0088	0.0146	0.0108	0.0021

1.5	MLE	Mean	0.5104	1.5404	0.5140	0.2507	0.5025	1.5405	0.5140	0.2477	0.5057	1.5277	0.5089	0.2498	0.5022	1.5136	0.5064	0.2499
		MSE	0.0130	0.0746	0.0027	0.0026	0.0105	0.0638	0.0027	0.0023	0.0069	0.0411	0.0015	0.0015	0.0054	0.0298	0.0011	0.0011
	MPSE	Mean	0.4711	1.4322	0.5090	0.2493	0.4689	1.4421	0.5090	0.2471	0.4812	1.4510	0.5045	0.2502	0.4846	1.4541	0.5022	0.2507
		MSE	0.0117	0.0670	0.0027	0.0026	0.0100	0.0569	0.0026	0.0023	0.0065	0.0382	0.0015	0.0015	0.0052	0.0291	0.0011	0.0011
	MSADE	Mean	0.6710	1.5919	0.4193	0.2929	0.6288	1.5798	0.4348	0.2816	0.5903	1.5454	0.4502	0.2751	0.5512	1.5115	0.4661	0.2672
		MSE	0.0933	0.1125	0.0156	0.0068	0.0652	0.0964	0.0135	0.0051	0.0356	0.0592	0.0075	0.0036	0.0166	0.0421	0.0041	0.0023
	MSALDE	Mean	0.5191	1.4783	0.4825	0.2618	0.5067	1.4810	0.4866	0.2565	0.5124	1.4815	0.4879	0.2582	0.5047	1.4717	0.4905	0.2562
		MSE	0.0168	0.0796	0.0036	0.0036	0.0139	0.0661	0.0037	0.0030	0.0096	0.0475	0.0022	0.0020	0.0072	0.0360	0.0016	0.0015
	LSE	Mean	0.5217	1.5047	0.4922	0.2599	0.5100	1.5106	0.4946	0.2550	0.5120	1.5066	0.4945	0.2554	0.5084	1.4937	0.4971	0.2552
		MSE	0.0155	0.0786	0.0035	0.0035	0.0126	0.0764	0.0033	0.0030	0.0080	0.0489	0.0021	0.0020	0.0063	0.0363	0.0016	0.0016
	WLSE	Mean	0.5169	1.5199	0.5013	0.2558	0.5064	1.5247	0.5035	0.2515	0.5083	1.5164	0.5015	0.2526	0.5057	1.5029	0.5025	0.2527
		MSE	0.0147	0.0749	0.0031	0.0031	0.0119	0.0709	0.0030	0.0027	0.0074	0.0456	0.0018	0.0018	0.0058	0.0327	0.0013	0.0013
	CME	Mean	0.5148	1.5540	0.5143	0.2517	0.5034	1.5540	0.5139	0.2476	0.5072	1.5371	0.5084	0.2500	0.5048	1.5154	0.5072	0.2512
		MSE	0.0164	0.0960	0.0040	0.0035	0.0132	0.0919	0.0038	0.0032	0.0082	0.0557	0.0023	0.0020	0.0065	0.0394	0.0018	0.0016
	ADE	Mean	0.5464	1.5678	0.4507	0.2601	0.5481	1.5408	0.4350	0.2639	0.5640	1.4898	0.4107	0.2755	0.5674	1.4490	0.4016	0.2820
		MSE	0.0148	0.0676	0.0049	0.0026	0.0124	0.0530	0.0066	0.0024	0.0102	0.0289	0.0094	0.0020	0.0092	0.0221	0.0106	0.0020

**Table 4:** The length of bootstrap CI for stress-strength reliability with different level of intervals: Case 1.

*θ*_1_		0.1						3					
*θ*_2_=0.1, *β*=0.5		90%		95%		99%		90%		95%		99%	
*n*, *m*		L.BP	L.BCP	L.BP	L.BCP	L.BP	L.BCP	L.BP	L.BCP	L.BP	L.BCP	L.BP	L.BCP
30, 40	MLE	0.00657	0.00658	0.00764	0.00764	0.01029	0.01030	0.00129	0.00128	0.00151	0.00152	0.00201	0.00200
	MPSE	0.00623	0.00628	0.00748	0.00744	0.01001	0.00991	0.00123	0.00124	0.00147	0.00147	0.00204	0.00204
	MSADE	0.00704	0.00708	0.00844	0.00846	0.01115	0.01130	0.00407	0.00403	0.00495	0.00494	0.00681	0.00667
	MSALDE	0.00707	0.00707	0.00850	0.00845	0.01070	0.01073	0.00166	0.00167	0.00201	0.00201	0.00262	0.00273
	LSE	0.00668	0.00676	0.00794	0.00781	0.01063	0.01085	0.00165	0.00164	0.00202	0.00203	0.00256	0.00258
	WLSE	0.00674	0.00680	0.00812	0.00811	0.01034	0.01034	0.00164	0.00163	0.00191	0.00191	0.00249	0.00250
	CME	0.00744	0.00744	0.00900	0.00900	0.01154	0.01158	0.00158	0.00158	0.00195	0.00196	0.00255	0.00256
	ADE	0.00620	0.00622	0.00764	0.00758	0.00987	0.00978	0.00218	0.00217	0.00255	0.00259	0.00332	0.00344

35, 45	MLE	0.00586	0.00592	0.00724	0.00718	0.00948	0.00943	0.00118	0.00117	0.00142	0.00141	0.00184	0.00185
	MPSE	0.00629	0.00607	0.00735	0.00744	0.00974	0.00979	0.00122	0.00122	0.00146	0.00146	0.00185	0.00188
	MSADE	0.00690	0.00697	0.00832	0.00838	0.01165	0.01135	0.00390	0.00394	0.00473	0.00480	0.00610	0.00653
	MSALDE	0.00674	0.00669	0.00786	0.00782	0.00982	0.00993	0.00167	0.00169	0.00202	0.00203	0.00252	0.00252
	LSE	0.00668	0.00668	0.00790	0.00790	0.01061	0.01061	0.00151	0.00150	0.00177	0.00178	0.00233	0.00232
	WLSE	0.00621	0.00626	0.00770	0.00772	0.01054	0.01081	0.00147	0.00145	0.00170	0.00172	0.00215	0.00216
	CME	0.00676	0.00680	0.00836	0.00839	0.01140	0.01133	0.00143	0.00141	0.00170	0.00169	0.00224	0.00226
	ADE	0.00574	0.00575	0.00674	0.00674	0.00875	0.00877	0.00206	0.00209	0.00245	0.00244	0.00341	0.00347

50, 60	MLE	0.00505	0.00500	0.00598	0.00598	0.00814	0.00821	0.00108	0.00108	0.00125	0.00125	0.00161	0.00162
	MPSEE	0.00484	0.00472	0.00585	0.00586	0.00815	0.00796	0.00109	0.00109	0.00126	0.00126	0.00163	0.00163
	MSADE	0.00618	0.00620	0.00740	0.00737	0.00931	0.00933	0.00297	0.00302	0.00369	0.00365	0.00465	0.00482
	MSALDE	0.00569	0.00568	0.00677	0.00681	0.00910	0.00908	0.00136	0.00134	0.00161	0.00163	0.00216	0.00220
	LSE	0.00552	0.00547	0.00650	0.00652	0.00887	0.00884	0.00142	0.00142	0.00166	0.00166	0.00211	0.00211
	WLSE	0.00558	0.00558	0.00681	0.00685	0.00947	0.00936	0.00124	0.00123	0.00148	0.00149	0.00211	0.00207
	CME	0.00597	0.00600	0.00694	0.00697	0.00908	0.00901	0.00131	0.00133	0.00163	0.00162	0.00211	0.00209
	ADE	0.00442	0.00438	0.00534	0.00517	0.00686	0.00689	0.00206	0.00203	0.00235	0.00236	0.00322	0.00322

70, 80	MLE	0.00405	0.00416	0.00501	0.00497	0.00646	0.00659	0.00087	0.00087	0.00105	0.00106	0.00146	0.00146
	MPSE	0.00407	0.00412	0.00488	0.00488	0.00669	0.00662	0.00088	0.00091	0.00108	0.00108	0.00143	0.00153
	MSADE	0.00512	0.00513	0.00623	0.00628	0.00807	0.00807	0.00191	0.00192	0.00230	0.00230	0.00310	0.00311
	MSALDE	0.00480	0.00483	0.00598	0.00596	0.00787	0.00798	0.00108	0.00110	0.00130	0.00129	0.00168	0.00168
	LSE	0.00465	0.00464	0.00557	0.00555	0.00797	0.00791	0.00108	0.00107	0.00128	0.00128	0.00181	0.00178
	WLSE	0.00463	0.00461	0.00551	0.00551	0.00762	0.00774	0.00100	0.00101	0.00123	0.00120	0.00153	0.00153
	CME	0.00492	0.00484	0.00585	0.00583	0.00782	0.00781	0.00110	0.00111	0.00129	0.00130	0.00160	0.00160
	ADE	0.00382	0.00378	0.00449	0.00449	0.00579	0.00575	0.00177	0.00178	0.00217	0.00219	0.00284	0.00280

**Table 5:** The length of bootstrap CI for stress-strength reliability with different level of intervals: Case 2.

*θ*_2_		0.4						2					
*θ*_1_=3, *β*=0.9		90%		95%		99%		90%		95%		99%	
*n*, *m*		L.BP	L.BCP	L.BP	L.BCP	L.BP	L.BCP	L.BP	L.BCP	L.BP	L.BCP	L.BP	L.BCP
30, 40	MLE	0.00321	0.00320	0.00393	0.00396	0.00493	0.00493	0.00652	0.00653	0.00765	0.00767	0.01011	0.01049
	MPSE	0.00326	0.00327	0.00392	0.00393	0.00537	0.00530	0.00644	0.00642	0.00763	0.00758	0.01048	0.01050
	MSADE	0.00689	0.00684	0.00805	0.00807	0.01032	0.01012	0.00726	0.00723	0.00875	0.00883	0.01161	0.01170
	MSALDE	0.00406	0.00408	0.00497	0.00497	0.00655	0.00642	0.00686	0.00687	0.00831	0.00833	0.01064	0.01064
	LSE	0.00373	0.00376	0.00443	0.00442	0.00575	0.00575	0.00706	0.00711	0.00867	0.00858	0.01125	0.01131
	WLSE	0.00361	0.00362	0.00427	0.00429	0.00563	0.00562	0.00679	0.00679	0.00793	0.00794	0.01074	0.01076
	CME	0.00369	0.00369	0.00443	0.00464	0.00607	0.00605	0.00721	0.00715	0.00834	0.00837	0.01101	0.01104
	ADE	0.00404	0.00396	0.00482	0.00483	0.00606	0.00622	0.00585	0.00585	0.00735	0.00733	0.00975	0.00966

35, 45	MLE	0.00312	0.00311	0.00370	0.00373	0.00444	0.00443	0.00564	0.00575	0.00689	0.00688	0.00839	0.00836
	MPSE	0.00320	0.00311	0.00374	0.00370	0.00487	0.00492	0.00579	0.00592	0.00716	0.00710	0.00955	0.00976
	MSADE	0.00617	0.00616	0.00723	0.00719	0.00952	0.00941	0.00668	0.00669	0.00851	0.00842	0.01074	0.01069
	MSALDE	0.00361	0.00359	0.00406	0.00419	0.00583	0.00601	0.00646	0.00639	0.00745	0.00752	0.00945	0.00949
	LSE	0.00354	0.00354	0.00428	0.00428	0.00578	0.00578	0.00664	0.00644	0.00787	0.00781	0.01064	0.01079
	WLSE	0.00358	0.00356	0.00425	0.00428	0.00538	0.00559	0.00638	0.00621	0.00734	0.00740	0.01003	0.01014
	CME	0.00363	0.00364	0.00424	0.00425	0.00570	0.00573	0.00700	0.00707	0.00835	0.00827	0.01122	0.01164
	ADE	0.00384	0.00383	0.00486	0.00487	0.00582	0.00595	0.00575	0.00575	0.00701	0.00699	0.00932	0.00919

50, 60	MLE	0.00250	0.00248	0.00302	0.00305	0.00446	0.00446	0.00496	0.00495	0.00598	0.00599	0.00810	0.00813
	MPSE	0.00251	0.00251	0.00306	0.00306	0.00418	0.00418	0.00515	0.00514	0.00626	0.00624	0.00824	0.00822
	MSADE	0.00489	0.00490	0.00583	0.00584	0.00781	0.00778	0.00552	0.00550	0.00660	0.00658	0.00878	0.00891
	MSALDE	0.00332	0.00330	0.00388	0.00394	0.00525	0.00533	0.00549	0.00549	0.00648	0.00649	0.00852	0.00853
	LSE	0.00321	0.00313	0.00389	0.00392	0.00509	0.00515	0.00520	0.00517	0.00612	0.00609	0.00771	0.00775
	WLSE	0.00286	0.00285	0.00342	0.00341	0.00481	0.00474	0.00514	0.00516	0.00633	0.00620	0.00801	0.00799
	CME	0.00311	0.00312	0.00370	0.00369	0.00484	0.00480	0.00574	0.00564	0.00687	0.00687	0.00879	0.00883
	ADE	0.00350	0.00353	0.00421	0.00410	0.00547	0.00550	0.00483	0.00478	0.00582	0.00584	0.00761	0.00756

70, 80	MLE	0.00227	0.00225	0.00267	0.00268	0.00363	0.00382	0.00407	0.00403	0.00510	0.00508	0.00658	0.00660
	MPSE	0.00225	0.00225	0.00266	0.00265	0.00340	0.00340	0.00391	0.00384	0.00479	0.00489	0.00638	0.00633
	MSADE	0.00353	0.00351	0.00427	0.00423	0.00583	0.00572	0.00516	0.00515	0.00644	0.00632	0.00843	0.00835
	MSALDE	0.00262	0.00265	0.00317	0.00317	0.00424	0.00427	0.00489	0.00489	0.00558	0.00569	0.00820	0.00817
	LSE	0.00274	0.00277	0.00335	0.00328	0.00441	0.00433	0.00472	0.00472	0.00565	0.00559	0.00740	0.00727
	WLSE	0.00263	0.00262	0.00314	0.00322	0.00394	0.00399	0.00482	0.00482	0.00581	0.00576	0.00689	0.00697
	CME	0.00281	0.00284	0.00320	0.00324	0.00439	0.00422	0.00462	0.00465	0.00558	0.00564	0.00781	0.00803
	ADE	0.00309	0.00299	0.00367	0.00366	0.00499	0.00516	0.00382	0.00378	0.00445	0.00440	0.00573	0.00585

**Table 6:** The length of bootstrap CI for stress-strength reliability with different level of intervals: Case 3.

*θ*_2_		0.4						1.5					
*θ*_1_=0.5, *β*=0.5		90%		95%		99%		90%		95%		99%	
*n*, *m*		L.BP	L.BCP	L.BP	L.BCP	L.BP	L.BCP	L.BP	L.BCP	L.BP	L.BCP	L.BP	L.BCP
30, 40	MLE	0.00588	0.00587	0.00712	0.00713	0.01018	0.01017	0.00529	0.00538	0.00620	0.00625	0.00885	0.00870
	MPSE	0.00619	0.00630	0.00748	0.00751	0.00925	0.00927	0.00535	0.00546	0.00647	0.00644	0.00835	0.00801
	MSADE	0.00693	0.00701	0.00828	0.00838	0.01189	0.01070	0.00697	0.00688	0.00825	0.00832	0.01067	0.01074
	MSALDE	0.00698	0.00698	0.00802	0.00802	0.01058	0.01057	0.00613	0.00610	0.00707	0.00720	0.01045	0.01032
	LSE	0.00664	0.00664	0.00807	0.00807	0.01066	0.01065	0.00582	0.00581	0.00712	0.00697	0.00955	0.00984
	WLSE	0.00675	0.00674	0.00801	0.00805	0.01058	0.01032	0.00628	0.00628	0.00720	0.00717	0.00924	0.00916
	CME	0.00690	0.00686	0.00884	0.00879	0.01185	0.01149	0.00607	0.00606	0.00727	0.00727	0.01031	0.01032
	ADE	0.00651	0.00643	0.00792	0.00780	0.01031	0.01040	0.00530	0.00530	0.00625	0.00628	0.00866	0.00871

35, 45	MLE	0.00616	0.00607	0.00725	0.00712	0.00976	0.00971	0.00489	0.00489	0.00594	0.00594	0.00838	0.00838
	MPSE	0.00585	0.00589	0.00713	0.00715	0.01039	0.01052	0.00488	0.00489	0.00582	0.00582	0.00781	0.00781
	MSADE	0.00686	0.00686	0.00826	0.00821	0.01091	0.01102	0.00671	0.00665	0.00787	0.00782	0.01018	0.00997
	MSALDE	0.00676	0.00689	0.00840	0.00831	0.01060	0.01078	0.00557	0.00555	0.00648	0.00649	0.00898	0.00903
	LSE	0.00635	0.00637	0.00783	0.00782	0.01019	0.01021	0.00556	0.00561	0.00674	0.00673	0.00936	0.00934
	WLSE	0.00630	0.00641	0.00768	0.00769	0.00969	0.00968	0.00538	0.00540	0.00637	0.00641	0.00860	0.00850
	CME	0.00676	0.00670	0.00782	0.00793	0.01056	0.01075	0.00601	0.00593	0.00695	0.00702	0.00969	0.00873
	ADE	0.00579	0.00579	0.00675	0.00681	0.00975	0.00975	0.00472	0.00474	0.00574	0.00572	0.00747	0.00751

50, 60	MLE	0.00540	0.00547	0.00658	0.00632	0.00862	0.00848	0.00412	0.00411	0.00512	0.00511	0.00676	0.00683
	MPSE	0.00533	0.00535	0.00619	0.00613	0.00775	0.00768	0.00415	0.00410	0.00495	0.00497	0.00664	0.00656
	MSADE	0.00593	0.00593	0.00702	0.00704	0.00904	0.00901	0.00561	0.00550	0.00670	0.00668	0.00930	0.00906
	MSALDE	0.00583	0.00575	0.00666	0.00665	0.00947	0.00945	0.00462	0.00475	0.00569	0.00562	0.00797	0.00786
	LSE	0.00544	0.00543	0.00662	0.00666	0.00834	0.00874	0.00471	0.00468	0.00558	0.00557	0.00716	0.00709
	WLSE	0.00523	0.00524	0.00610	0.00611	0.00848	0.00847	0.00426	0.00422	0.00507	0.00508	0.00698	0.00698
	CME	0.00539	0.00540	0.00642	0.00630	0.00869	0.00866	0.00474	0.00477	0.00576	0.00581	0.00726	0.00745
	ADE	0.00462	0.00464	0.00538	0.00538	0.00773	0.00778	0.00385	0.00383	0.00472	0.00472	0.00592	0.00595

70, 80	MLE	0.00439	0.00439	0.00517	0.00517	0.00616	0.00616	0.00351	0.00360	0.00421	0.00423	0.00544	0.00593
	MPSE	0.00423	0.00425	0.00516	0.00512	0.00684	0.00692	0.00352	0.00348	0.00409	0.00410	0.00551	0.00555
	MSADE	0.00498	0.00481	0.00596	0.00585	0.00798	0.00797	0.00471	0.00480	0.00575	0.00569	0.00748	0.00749
	MSALDE	0.00478	0.00477	0.00583	0.00574	0.00773	0.00776	0.00418	0.00415	0.00499	0.00497	0.00664	0.00655
	LSE	0.00487	0.00479	0.00592	0.00589	0.00747	0.00737	0.00402	0.00402	0.00475	0.00475	0.00635	0.00634
	WLSE	0.00477	0.00478	0.00583	0.00592	0.00751	0.00750	0.00378	0.00378	0.00461	0.00461	0.00597	0.00597
	CME	0.00479	0.00478	0.00558	0.00559	0.00760	0.00760	0.00420	0.00423	0.00506	0.00496	0.00637	0.00636
	ADE	0.00378	0.00387	0.00461	0.00459	0.00589	0.00582	0.00335	0.00337	0.00400	0.00401	0.00505	0.00504

**Table 7:** MLEs, SEs, and KSS test with *P* value for breaking strengths of jute fiber data.

	*X*		*Y*	
	*θ*_1_	*β*_1_	*θ*_2_	*β*_2_
Estimate	483.9833	1.1803	228.9500	1.0849
SE	363.0840	0.1515	158.6915	0.1466
KSS	0.1708		0.1566	
*P* value	0.3092		0.4116	

**Table 8:** Estimation with SE and stress-strength reliability for breaking strengths of jute fiber data.

	MLE		MPSE		MSADE		MSALDE	
	Estimate	SE	Estimate	SE	Estimate	SE	Estimate	SE

*θ*_1_	441.8773	262.2884	451.5945	231.2679	483.9824	201.5166	483.8995	198.1652
*θ*_2_	315.0805	171.8451	274.3958	112.3324	228.9514	106.1569	229.0038	86.2184
*β*	1.1569	0.1147	1.1636	0.9557	1.0047	0.0154	1.1284	0.0955
*R*	0.5838		0.6220		0.6789		0.6788	

	LSE		WLSE		CME		ADE	
*θ*_1_	431.0775	316.5598	851.4172	175.4259	461.7079	319.4659	408.3478	465.6265
*θ*_2_	328.7274	716.6545	653.3498	130.7536	368.3411	416.8481	315.7379	341.9234
*β*	1.1359	0.4072	1.2752	0.0358	1.1497	0.4990	1.1339	0.2050
*R*	0.5674		0.5658		0.5562		0.5639	

**Table 9:** MLEs, SEs, and KSS test with *P* value for carbon fibers data.

	*X*		*Y*	
	*θ*_1_	*β*_1_	*θ*_2_	*β*_2_
Estimates	23.2675	4.1271	230.4763	5.4338
SE	5.7133	0.3382	110.9623	0.5081
KSS	0.1001		0.1336	
*P* value	0.5531		0.1700	

**Table 10:** Estimation with SE and stress-strength reliability for carbon fibers data.

	MLE			MSADE			LS		
	Estimates	SE	*R*	Estimates	SE	*R*	Estimates	SE	*R*

*θ*_1_	103.9460	30.2423	0.7722	223.4832	21.2861	0.7108	225.1419	434.1815	0.7456
*θ*_2_	30.6687	6.3435		90.9306	1.3112		76.8348	123.6565	
*β*	4.5745	0.2792		5.2734	0.0064		5.3519	1.7957	

	WLS			CVM			AD		
*θ*_1_	325.8187	29.0474	0.7626	252.1115	490.7954	0.7493	86.1874	51.9326	0.7119
*θ*_2_	101.4512	7.6739		84.3294	137.0736		34.8749	17.2440	
*β*	5.7352	0.0829		5.4569	1.8128		4.4401	0.5642	

## Data Availability

All the data are included in the manuscript.
